# Hepatocyte estrogen-related receptor α modulates a gluconeogenic–epigenetic crosstalk counteracting MASLD/MASH progression

**DOI:** 10.1038/s12276-026-01707-1

**Published:** 2026-05-08

**Authors:** Jun Gao, Meng Yang, Rui Duan, Tongling Huang, Pengda Li, Lu Gao, Zhaocheng Lu, Chi-Wai Wong, Chang-An Geng, Min Guan

**Affiliations:** 1 Institute of Biomedicine and Biotechnology, Shenzhen Institutes of Advanced Technology, Chinese Academy of Sciences, Shenzhen, China; 2 Guangdong Provincial Key Laboratory of Medical Immunology and Molecular Diagnostics, Institute of Aging Research, Guangdong Medical University, Dongguan, China; 3 School of Physical Education and Sports Science, South China Normal University, Guangzhou, China; 4 Guangzhou Huazhen Biosciences, Guangzhou, China; 5 State Key Laboratory of Phytochemistry and Plant Resources in West China, Kunming Institute of Botany, Chinese Academy of Sciences, Kunming, China

**Keywords:** Metabolic disorders, Metabolism, Transcriptional regulatory elements, Epigenetics

## Abstract

Lactate has been recognized as a major fuel substrate and also a lactyl-group donor for histone lysine lactylation. Hepatocytes act as lactate-consuming cells owing the high oxidative capability especially during exercise, a primary nonpharmacological intervention for alleviating metabolic dysfunction-associated steatotic liver diseases including steatohepatitis (MASLD/MASH). However, little is known regarding how lactate links the metabolic–epigenetic axis in hepatocytes. Here we show that declined estrogen-related receptor α (ESRRA) expression occur in MASLD/MASH accompanied with elevated levels of lactate and histone lactylation, particularly H3K18la. Such dysregulation can be partially rescued by chronic exercise in aged mice or exacerbated by genetic ablation of hepatocyte ESRRA. Mechanistically, exercise-induced ESRRA/PPARGC1A facilitates lactate consumption through transcriptional regulation of lactate dehydrogenase B and glucose-6-phosphatase catalytic subunit 1, rewiring lactate from a lactyl donor to gluconeogenic precursor in hepatocytes. Hepatocyte-specific ESRRA overexpression counteracts MASLD/MASH progression in mice, rectifying aberrant H3K18la accumulation and its marked gene transcripts that are involved in liver pathology. Our findings reveal that ESRRA functions as an exercise executor linking metabolism with epigenetic modification, highlighting a gluconeogenic–epigenetic regulatory axis that could be fine-tuned to mitigate risk factors of MASLD/MASH such as aging, menopause, a sedentary lifestyle and malnutrition.

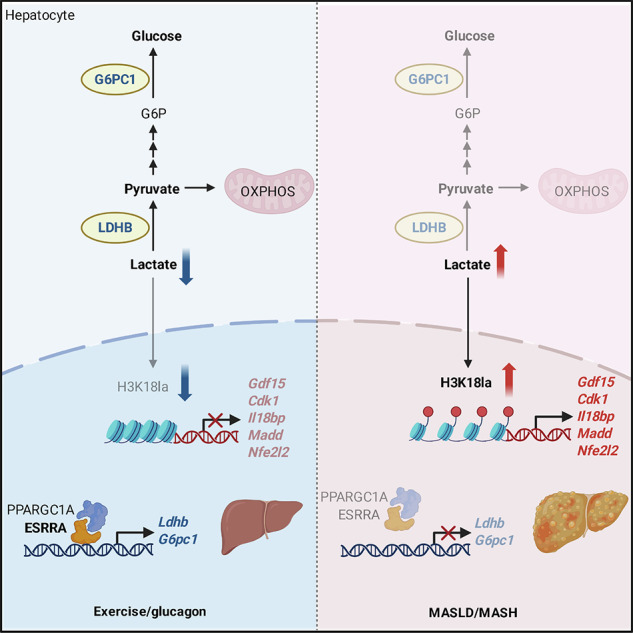

## Exercise modulates lactate’s role in liver epigenetics

Metabolic dysfunction-associated steatotic liver diseases (MASLD), including steatohepatitis, are prevalent conditions that can progress to severe liver complications. This study explores the role of the orphan nuclear receptor estrogen-related receptor alpha (ESRRA) in MASLD progression, particularly its interaction with lactate metabolism and histone lactylation. Using in vitro and in vivo models, the authors demonstrate that ESRRA modulates lactate clearance and gluconeogenesis in hepatocytes, which is crucial for preventing MASLD progression. They show that ESRRA deficiency leads to increased histone lactylation, exacerbating liver fibrosis and inflammation. The study highlights that ESRRA overexpression in hepatocytes reduces lactate-driven histone lactylation, offering a protective effect against MASLD. These findings suggest that targeting ESRRA could be a potential therapeutic strategy for MASLD, with exercise enhancing ESRRA’s beneficial effects.

This summary was initially drafted using artificial intelligence, then revised and fact-checked by the author.

## Introduction

Metabolic dysfunction-associated steatotic liver diseases including steatohepatitis (MASLD/MASH) is a prevalent chronic liver disease that can progress to cirrhosis and even hepatocellular carcinoma (HCC). The pathogenesis of MASLD is multifactorial and triggered by environmental factors such as hypercaloric nutrition and physical inactivity in the context of genetic predisposition^1^. In addition, an older age of ≥65 years is considered an independent risk factor for MASLD/MASH and related HCC development^2^. Notably, postmenopausal women among the aged population are prone to hepatic steatosis and MASH with advanced fibrosis in part owing to the lack of estrogen protection^3^. Thus, the metabolic disorder coupled with high risk factors such as sedentary lifestyle, menopause and aging contribute to a vicious pathogenic progression leading to MASLD/MASH and even HCC. However, it remains unclear whether these risk factors share common mechanisms underlying MASLD to MASH progression.

Clinical evidence strongly supports the role of proper physical exercise as a primary intervention combating MASLD and MASH^4^. Acute exercise metabolism involves interorgan coordination between multiple tissues, and the cumulative effects during regular chronic exercise induces tissue-specific adaptations, promoting whole-body health and preventing disease^5^. A healthy liver exhibits a higher net lactate clearance through gluconeogenesis than any other organ does, accounting for the majority of lactate consumption in the whole body and is particularly reinforced by exercise or fasting. On the contrary, elevation of blood lactate, that is hyperlactemia or lactic acidosis, correlates with illness or injury severity in patients with cancer, sepsis or liver failure^6^. Apart from being the major gluconeogenic precursor, lactate has been recently recognized as a major fuel source for the tricarboxylic acid (TCA) cycle, a signaling molecule or a posttranslational precursor that drives a novel epigenetic modification known as histone lactylation^7–9^. It has been recently reported that lactylation on histone H3 lysine 18 (H3K18la) driven by glycolysis in hepatic stellate cells (HSCs) promotes liver fibrosis^10^. Enrichment of lactylation on mitochondrial fission 1 protein or H3K18 occurs in patients with sepsis accompanied with high blood lactic acid^11,12^. Moreover, an integrative global lactylome and proteome analysis in a hepatitis B virus-related HCC cohort identifies a prevalent modification of lysine lactylation (Kla) on proteins that are particularly involved in metabolic pathways^13^. Distinct from the HSC or liver tumor cell that is a glycolytic lactate producer cell, the hepatocyte acts as the consumer cell owing the high oxidative capability of gluconeogenic flux to consume both hepatocellular and extrahepatic lactate. Opposite to lactate dehydrogenase A (LDHA), an increase of which gains glycolysis-produced lactate, lactate dehydrogenase B (LDHB) favors lactate catabolism that is necessary for lactate-derived gluconeogenesis in the liver. Nonetheless, LDHB has been largely overlooked owing to its quite lower expression in the liver compared with other tissues such as heart and skeletal muscle. Of note, a recent finding identifies that lactate is the prominent lipogenic substrate in the liver^14^. It is plausible that insufficient lactate catabolism might affect liver diseases progression. However, little is known about how lactate links the metabolic–epigenetic axis in hepatocytes, which may play potential roles in MASLD/MASH progression.

Orphan nuclear receptor estrogen-related receptor α (ESRRA; also known as ERRα or NR3B1) and its co-activator peroxisome proliferative activated receptor gamma co-activator 1 alpha (PPARGC1A) can transcriptionally regulate each other mutually and coordinately fine-tune the expression of numerous target genes involved in lipid and carbohydrate metabolism^15–17^. We previously found that hepatocyte ESRRA regulates sex disparity in lipid metabolism; moreover, adipocyte ESRRA mediates secreted factors that influence bone formation in a mice model resembling the postmenopausal or obese condition^18,19^. Importantly, ESRRA is an effector in response to metabolic stress, such as fasting, exercise, calorie restriction, overnutrition or cold exposure in a cell and/or tissue context-specific manner^20–23^. Here, we observe that ESRRA–PPARGC1A signaling is perturbed in MASLD/MASH livers induced by multiple risk factors, contributing to enrichment of histone lactylation, especially H3K18la. Using in vitro and in vivo approaches, we define ESRRA as an exercise effector, bridging a gluconeogenic and epigenetic crosstalk that requires hepatocyte LDHB. Furthermore, inhibition of H3K18la by hepatocyte-specific ESRRA overexpression results in decreased expression of H3K18la-marked genes that are involved in inflammation, fibrosis and tumorigenesis, conferring a protective effect against MASH development in mice. These findings highlight a previous unrecognized gluconeogenic–epigenetic mechanism in hepatocytes for combating chronic liver diseases.

## Methods

### Animal information

The generation method of *Esrra*^fl/fl^ mice was described in our previous literature^18^. The Albumin-Cre (Alb-Cre) transgenic mice were obtained from GemPharmatech (strain T003814). Hepatocyte-specific *Esrra*-knockout mice, referred to as *Esrra*^HKO^, were generated by crossing *Esrra*^fl/fl^ mice with Alb-Cre mice. Primers for genotyping are presented in Supplementary Table 1. *Ldhb*^fl/fl^ mice were purchased from GemPharmatech (strain T018265). In brief, *Ldhb*^fl/fl^ mice were generated using a CRISPR/Cas9-mediated genome engineering system. The loxP sites located at the front of exon 3 and the rear of exon 6 in the *Ldhb* gene are oriented in the same direction. Wild-type C57BL/6 mice were obtained from GemPharmatech.

For the age-related MASLD model, male mice at 18 or 25 months of age were used to study spontaneous liver steatosis. For the overiectomy (OVX) model, 8-week-old female mice underwent bilateral ovariectomy and were killed 8 weeks after surgery to mimic the postmenopausal condition. For the diet-induced MASLD model, 5-week-old female mice were fed a high‑fat, high‑cholesterol (HFHC) diet (Research Diets, D09100301) containing 40 kcal% fat (18% trans fat), 22 kcal% fructose and 2% cholesterol for 28 weeks, as described in our previous study^18^. For the MASH model, 6- or 9-week-old male mice were fed a Gubra-Amylin nonalcoholic steatohepatitis (GAN) diet (Research Diets, #D09100310) containing 40 kcal% fat (mostly palm oil), 22 kcal% fructose and 2% cholesterol together with carbon tetrachloride (CCl_4_) (Macklin, #C805329) for 12 weeks to closely mimic the features of human MASH^24^. CCl_4_ was administered intraperitoneally twice weekly at a dosage of 0.2 µl/g body weight. For high-fat diet (HFD)-fed mice, 6-week-old male mice were fed an HFD (Research Diets, #D12492) containing 60 kcal% fat, 20 kcal% protein and 20 kcal% carbohydrates for 12 weeks. All mice were maintained in a temperature- and humidity-controlled environment on 12 h light–dark cycles with free access to food and water. Mice were killed via cervical dislocation following anesthesia induced by isoflurane, and the samples were subsequently collected for further analysis. Alanine aminotransferase (ALT; Solarbio, #BC1555), aspartate aminotransferase (AST; Solarbio, #BC1565), triglycerides (TG; Applygen, #E1025) and total cholesterol (TC; Applygen, #E1015) from blood or liver samples were measured using commercial kits according to the manufacturer’s instructions. The experimental protocols for mice were reviewed and approved by the Ethical Committee at Shenzhen Institute of Advanced Technology, Chinese Academy of Sciences.

### LTT and lactic acidosis assay

For the lactate tolerance test (LTT), 16-h-fasted mice were intraperitoneally injected with sodium lactate (Lac; Aladdin, #S108837) at a dose of 2 mg/g body weight. For the lactic acidosis experiment, mice received intraperitoneal injections of L-lactic acid (LA; Aladdin, #L122089) at a dose of 0.5 mg/g body weight and then the number of mice surviving within 120 h was recorded. Blood glucose and lactate were measured using a glucometer (Roche Diagnostics) and blood lactate meter (EKF Diagnostics).

### Exercise protocol and measurement

A total of 3 days before the treadmill experiments, mice were acclimatized to treadmill running for 8 min per day at a speed of 10 cm/s at a 0° inclination. For treadmill exercise, mice were fasted for 6 h and then ran on a treadmill with a 10° inclination at a speed of 10 cm/s for the first 3 min. The speed was increased by 2 cm/s every 2 min until the speed reached 38 cm/s. Blood glucose and lactate were monitored during exercise. For the exercise capacity test, the speed was accelerated to 46 cm/s as previously described and kept at 46 cm/s until the mice were exhausted. Exhaustion was defined as the mice refusing to exercise, and then the time to exhaustion and total running distance were recorded. For moderate-intensity continuous exercise in aged mice, the experiment protocol was described in our previous literature^25^. In brief, mice ran on a treadmill with an inclination of 0° at 60% of the maximal running speed (30 m/min) for 42 min, and 20-month-old mice participated in the exercise training every 2 days until 25 months of age.

### Histology and immunofluorescence staining

Paraffin-embedded liver sections were subjected to hematoxylin and eosin (H&E) staining (Beyotime, #C0105S), Sirius Red staining (Solarbio, #G1472) and Periodic Acid-Schiff (PAS) staining (Solarbio, #G1281) according to the manufacturer’s instructions. OCT-embedded liver sections were applied to Oil Red O staining (Solarbio, #G1261). For immunofluorescence staining, the liver sections were incubated overnight at 4 °C with primary antibodies F4/80 (Abcam, ab6640, 1:200) or CD11c (Abcam, ab11029, 1:200). Then, the liver sections were incubated with secondary antibodies Alexa Fluor 555-conjugated goat anti-rat IgG (Abcam, #ab150158, 1:200), Alexa Fluor 555-conjugated goat anti-mouse IgG (Abcam, #ab150114, 1:200) or Alexa Fluor 488-conjugated goat anti-rabbit IgG (Abcam, #ab150077, 1:200) for 1 h. The quantification of Sirius Red-, Oil Red O-, PAS-, F4/80- and CD11c-stained areas was calculated using ImageJ software.

### Cell culture

Primary hepatocytes were isolated from 8–12-week-old male mice. Livers were perfused with Hank’s buffered saline solution (HBSS) through the portal vein, followed by the second perfusion with HBSS containing 4 mmol/l CaCl_2_ and 0.5 mg/ml collagenase type II (Meilunbio, #MB2665). After perfusion, livers were dissected and filtered through a 70-μm cell strainer (Corning, #352350). Following removal of cell debris by density gradient centrifugation using Percoll (Biorigin, #BN31708), hepatocytes were plated on dishes coated with rat-tail collagen type I (Corning, #354236) and cultured in Dulbecco’s modified Eagle’s medium (DMEM; Gibco, #C11995500BT) containing 4.5 g/l glucose, 10% fetal bovine serum (FBS, TransGen Biotech, #FS301-02), 1% penicillin–streptomycin, 100 nM dexamethasone (MCE, #HY-14648), 1 nM insulin (Yeasen, #40112ES25) and 10 mM HEPES. After 6 h of culture, the medium was replaced by DMEM (Hyclone, #SH30021.01) containing 1 g/l glucose, 10% FBS, 1% penicillin–streptomycin and 10 mM HEPES. Hepatocytes were treated with 10 mM lactate in the presence or absence of 100 µM C646 (MCE, #HY-13823) for 24 h before examining histone lactylation. Huh7 cells (SCSP-526) were obtained from the Chinese Academy of Sciences Cell Bank and cultured in DMEM containing 4.5 g/l glucose, 10% FBS and 1% penicillin–streptomycin at 37 °C and 5% CO_2_.

### Glucose output assay

Primary hepatocytes were cultured in glucose-free DMEM (Gibco, #A14430-01) supplemented with 0.5% fatty acid-free BSA (Yeasen, #36104ES25), 10 mM HEPES and 10 mM Lac with or without 1 mM sodium pyruvate (Pyr; Sigma, #P2256). For glucose output assay, hepatocytes were treated with some of the following agents: 20 µM C29 (ChemPartner, #S1039), 20 µM H89 (Selleck, #S1582), 100 nM glucagon (MCE, #HY-P0082), 50 μM Bt_2_-cAMP (Sigma, #D0627), 5 mM α-CHCA (Selleck, #S8612) or 5 µM UK5099 (Selleck, #S5317). After incubation for 6 h, glucose in medium was examined using the Glucose Assay Kit (Applygen, #E1010-200) per the manufacturer’s instruction.

### OCR measurement

Hepatocytes were seeded in XF96 cell culture plates at a density of 10,000 cells per well. After infection with indicated adenoviruses or incubation with 5 μM UK5099, hepatocytes were cultured in XF base medium (Agilent, #103575-100) supplemented with 10 mM Lac but without glucose, glutamine and pyruvate at 37 °C in a CO_2_-free incubator for 1 h. The injections of oligomycin (1.5 μM), carbonyl cyanide-*p*-trifluoromethoxyphenylhydrazone (1 μM), and rotenone/antimycin A (0.5 μM) were performed during continuous oxygen measurements. Oxygen consumption rate (OCR) was measured using an XF96e Extracellular Flux Analyzer (Agilent Technologies) and normalized to the total protein (TP) content of each sample.

### Plasmid constructs and luciferase assays

*Esrra* and *Ppargc1a* were inserted into pcDNA4 vector as in our previous literature^18^. The promoter regions of *Ldhb* and *G6pc1* were cloned into pGL3-Basic luciferase reporter vectors, generating pGL3-*Ldhb*-WT-luc and pGL3-*G6pc1*-WT-luc. The putative ESRRA binding sites A, B and C on *G6pc1* promoter was mutated to generate pGL3-*G6pc1*-mutA-luc, pGL3-*G6pc1*-mutB-luc and pGL3-*G6pc1*-mutC-luc. DNA sequencing confirmed the accuracy of all constructs and mutations. Primers used to generate plasmid constructs are presented in Supplementary Table 2.

Huh7 cells were seeded into cell culture plates and cultured in phenol red-free DMEM (Gibco #A1048801) containing 10% charcoal stripped FBS. The cells were transfected with promoter-luciferase reporters and together with expression vectors using lipofectamine 3000 reagent (Invitrogen, #L3000015). Cells were then treated with 20 μM C29 or dimethylsulfoxide for 24 h and then were lysed for measurement of luciferase activity using the Dual-Luciferase Reporter Assay System (Promega, #E1960). Renilla luciferase was used to normalize the transfection efficiency.

### ChIP

Chromatin immunoprecipitation (ChIP) assays were carried out using ChIP assay kit (CST, #9005S) according to the manufacturer’s instruction. Samples were fixed with 1% formaldehyde and quenched with glycine solution. Subsequently, nuclei were extracted and digested with micrococcal nuclease. The extracts containing 10 μg cross-linked chromatin were immunoprecipitated with ESRRA (CST, #13826S) or IgG antibodies (CST, #2729) and captured with protein G magnetic beads (CST, #9006). DNA was purified using spin columns and analyzed using quantitative PCR (qPCR). Primer sequences for ChIP–qPCR are presented in Supplementary Table 3. The quantity of immunoprecipitated DNA for each sample was normalized to IgG-associated DNA and shown as relative fold enrichment.

### Adenovirus production and infection

The generation of Ad-ESRRA was described in our previous literature^18^. Ad-Cre, Ad-LDHB and Ad-G6PC1 were obtained from Obio Technology. In brief, cDNA encoding *Cre, Ldhb* or *G6pc1* was inserted into a shuttle vector driven by the cytomegalovirus promoter, and the AdEasy system was used to build the virus. Adenoviruses were multiplied in HEK-293 cells and purified by CsCl density gradient ultracentrifugation. Subsequently, adenoviruses were injected intravenously into mice at a dose of 1 × 10^9^ plaque-forming units per mouse or infected hepatocytes at a dose of 20 plaque-forming units per cell.

### AAV production and infection

To generate the AAV8-TBG-ESRRA, *Esrra* cDNA was inserted into a pAAV8-TBG-MCS-WPRE vector and driven by a thyroxin-binding globulin (TBG) promoter. Adeno-associated viruses (AAVs) were purified by density gradient ultracentrifugation and their titers were ascertained through qPCR. AAVs were injected into the mice via tail vein at a dose of 1 × 10^11^ viral genomes/mouse. In the age-related MASLD model, male mice were injected with AAVs at 12 months of age and killed at 18 months for analysis. In the MASH model, male mice were injected with AAVs at 6 weeks of age and killed 15 weeks later for analysis.

### CUT&Tag and data analysis

Nuclei extracted from liver tissues were washed with buffer solution (20 mM HEPES, 150 mM NaCl, 0.5 mM spermidine, 1× protease inhibitor cocktail) and then incubated with concanavalin A-coated magnetic beads. Subsequently, bead-bound nuclei were resuspended with DIG wash buffer (20 mM HEPES, 150 mM NaCl, 0.5 mM spermidine, 1× protease inhibitor cocktail, 0.05% digitonin, 2 mM EDTA). The bead-bound nuclei were incubated with a primary antibody against H3K18la (PTM, #1427RM) or an IgG control antibody (Millipore, #12-370) on a rotating platform overnight at 4 °C. The secondary anti-rabbit IgG antibody (Millipore, #AP132) was then added and incubated for 1 h. The nuclei were washed in DIG wash buffer and incubated with pA-Tn5 adapter complex for 1 h. Thereafter, nuclei were washed in DIG-MED buffer (0.01% digitonin, 20 mM HEPES, 300 mM NaCl, 0.5 mM spermidine, 1× protease inhibitor cocktail) and incubated in tagmentation buffer (10 mM MgCl2 in DIG-MED Buffer) for 1 h at 37 °C. DNA fragmentation was then purified using phenol–chloroform–isoamyl alcohol extraction and ethanol precipitation. The Cleavage Under Targets and Tagmentation (CUT&Tag) library was generated using a PCR system and sequenced on the Illumina Novaseq 6000 platform (LC Bio Technology). Clean reads were obtained from raw reads by filtering out adapters, short-fragment reads and other low-quality reads. The filtered reads were aligned to the reference mouse genome assembly (mm10) using the BWA program. Peak Calling was performed using MACS2 software and a *q* value < 0.05 was used as the selection threshold. BEDTools was used to calculate the distance from each peak to the nearest gene and to perform peak annotation. Peaks located within ±3 kb of the transcription start site were defined as promoter-associated peaks. We also applied a permutation-based peak–gene linking analysis in which H3K18la peak locations were randomly shuffled (10,000 iterations) while preserving chromosome-level peak density. The false discovery rate (adjusted *P* value) was determined using the Benjamini–Hochberg procedure for multiple-comparison adjustment. The differential peaks were assessed using the DESeq2 algorithm with a false discovery rate <0.05 and were subjected to enrichment analysis of Gene Ontology (GO) functions and Kyoto Encyclopedia of Genes and Genomes (KEGG) pathways. Significantly enriched KEGG pathways and GO terms were chosen according to *P* < 0.05. Differential peaks were visualized using IGV version 2.17.4.

### RNA isolation and RT–qPCR

For quantitative PCR with reverse transcription (RT–qPCR) analysis, total RNA was extracted using TRIzol reagents (Accurate Biology, #AG21101) and reverse-transcribed into complementary DNA using a HiScript III 1st Strand cDNA Synthesis Kit (Vazyme #R312). RT–qPCR was performed using RealStar Green Fast Mixture (Genstar, #A301-10) and relative mRNA levels were normalized to β-actin or *18S rRNA*. The primers for RT–qPCR are presented in Supplementary Table 4.

### RNA sequencing and data analysis

RNA concentration and integrity were evaluated using the Qubit RNA Assay Kit in Qubit 2.0 Flurometer (Life Technologies), RNA Nano 6000 Assay Kit and the Bioanalyzer 2100 system (Agilent Technologies). Sequencing libraries were generated using the RNA Library Prep Kit (NEB, #E7770) and then sequenced on an Illumina Novaseq 6000 platform (LC Bio Technology). DEGs were determined using the DESeq2 algorithm and then were performed enrichment analysis of GO functions and KEGG pathways. GO terms and KEGG pathways with significant enrichment were chosen based on *P* < 0.05.

### Protein extraction and western blot analysis

For histone extraction, livers or hepatocytes were homogenized in lysis buffer (0.5% NP-40, 1 mM EDTA, 20 mM Tris, 500 mM NaCl, and 1× protease inhibitor cocktail), then centrifuged to obtain the nuclear pellet, after which histones were extracted by dissolving in 0.2 M HCl. TP from livers or hepatocytes was extracted with RIPA lysis buffer (Beyotime, #P0013B) containing protease inhibitor cocktail. The concentration of protein was measured using a bicinchoninic acid (BCA) Protein Assay Kit (Thermo Fisher, #23227). Protein lysates were separated by SDS–polyacrylamide gel electrophoresis (SDS–PAGE) and transferred onto polyvinylidene difluoride (PVDF) membranes (Millipore, #ISEQ00010). Membranes were blocked with Tris-buffered saline containing 0.1% Tween-20 (Sangon, #A600560) and 5% nonfat milk (Sangon, #A600669). The blots were probed with primary antibodies against ESRRA (CST, #13826S, 1:1000), G6PC1 (Abcepta, #AP5224c, 1:1000), LDHB (Abcam, #Ab75167, 1:2000), LDHB (Proteintech, #14824-1-AP, 1:5000), LDHA (Proteintech, #19987-1-AP, 1:5000), PPARGC1A (Millipore, #AB3242, 1:1000), tubulin (BPI, #AbM59005-37B-PU, 1:10000), H3K18la (PTM, #1406RM, 1:1000), H3K9la (PTM, #1419RM, 1:1000), H3K14la (PTM, #1414RM, 1:1000), H3K27la (PTM, #1428, 1:1000), H3 (Proteintech, #17168-1-AP, 1:2000) and Pan Kla (PTM, #1401, 1:1000). Blots were incubated with a horseradish peroxidase-conjugated goat anti-rabbit antibody (EarthOx Life Science, #E030120-02, 1:10000) or a goat anti-mouse antibody (EarthOx Life Science, #E030110-02, 1:10000). Subsequently, protein was visualized using a chemiluminescence kit (Millipore, #WBLUR0500).

### LDH activity assay

Lactate-to-pyruvate lactate dehydrogenase (LDH) activity was determined using an LDH activity assay kit (Solarbio, #BC0685) per the manufacturer’s protocols. In brief, hepatocytes or liver samples were homogenized at 4 °C and then the supernatant was collected to examine LDH activity according to the absorbance at 450 nm of the 2,4-dinitrophenylhydrazone that formed from pyruvate .

### Measurement of lactate content

The lactate levels were measured using a lactate assay kit (Jiancheng Bioengineering Institute, #A019-2-1) according to the manufacturer’s instructions. Livers or hepatocytes were lysed at 4 °C and then the supernatant was collected. Lactate levels were determined according to the absorbance of the sample at 530 nm and normalized against the TP content of each sample.

### Statistical analysis

To determine whether nonparametric or parametric tests were used, animal experimental datasets were measured for normality using the Shapiro–Wilk test (reject threshold defined as *P* < 0.05). For comparisons between two groups, a two-tailed Student’s *t*-test was used to analyze statistical significance as indicated in the figure legends. For multiple comparisons, one-way analysis of variance (ANOVA) with Tukey’s post hoc test and two-way ANOVA with Tukey’s post hoc test were used to evaluate statistical significance as noted in the figure legends. The results are presented as mean ± s.d. Significance was determined by the following *P* values: n.s., no significance; *P < 0.05; **P < 0.01; and ***P < 0.001. GraphPad Prism 8.0 was applied to perform statistical analyses.

## Results

### Elevated levels of lactate and histone lactylation in hepatosteatosis and liver fibrosis induced by diverse high risk factors are inversely linked to ESRRA/PPARGC1A expression

**Fig. 1:**
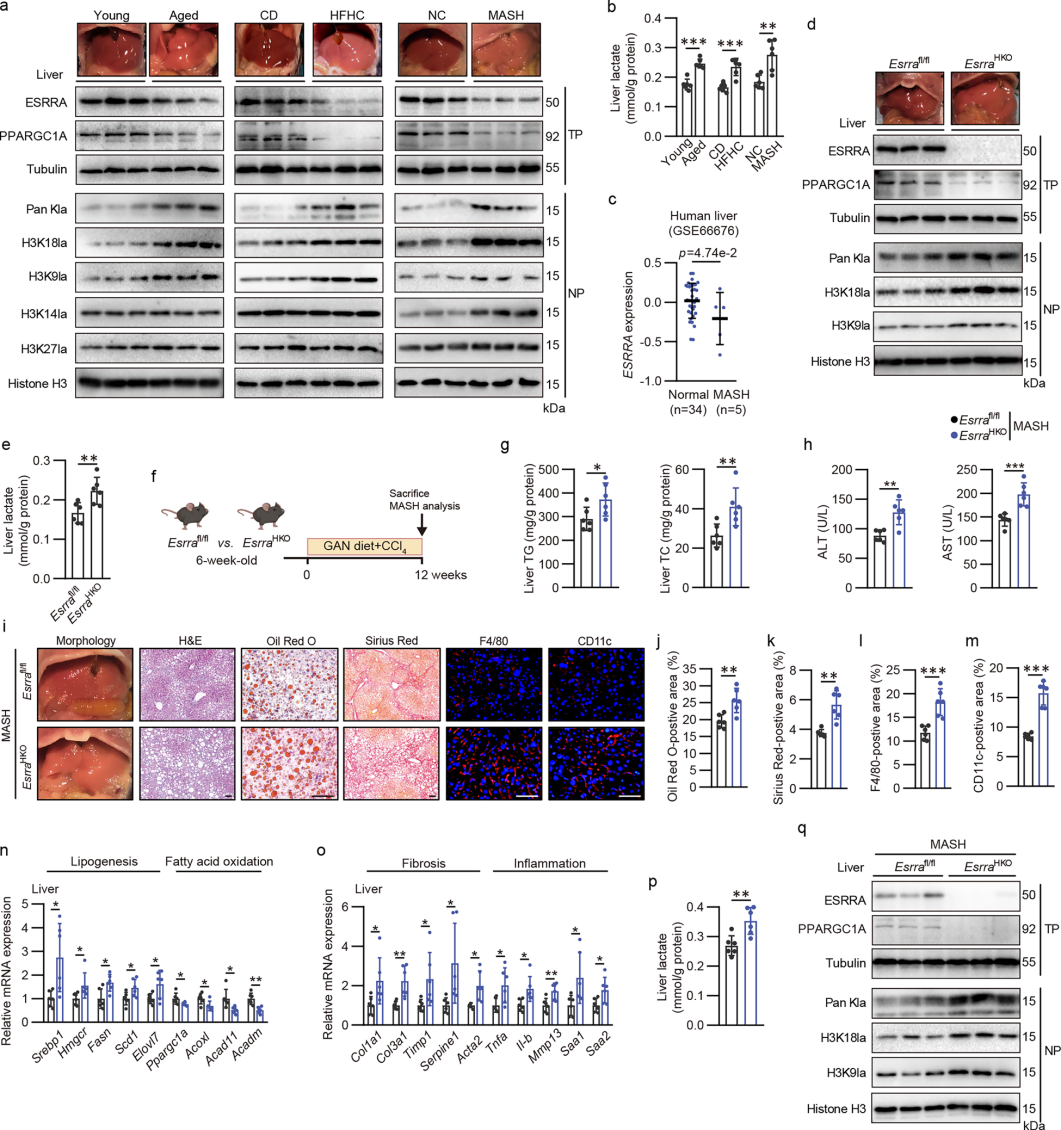
Declined hepatic ESRRA/PPARGC1A expression and accumulated histone lacylation are associated with MASLD/MASH induced by diverse risk factors, and hepatocyte-specific ESRRA ablation exaggerates MASH progression in mice. **a** Gross morphology of livers from young (3-month-old) and aged (25-month-old) male mice; from female mice fed a CD or an HFHC diet for 28 weeks; and from male MASH mice challenged with a GAN diet plus CCl_4_ for 12 weeks, together with male NC mice. Western blots showing indicated hepatic proteins from 16-h-fasted mice. Tubulin served as internal control for TP, and nuclear proteins were normalized to histone H3. **b** Hepatic lactate levels in **a**. *n* = 6 mice. **c** *ESRRA* mRNA levels in patients with normal and MASH livers from GSE66676 datasets. **d** Protein levels of ESRRA, PPARGC1A, Pan kla, H3K18la and H3K9la in *Esrra*^HKO^ and Esrra^fl/fl^ mice livers. **e** Hepatic lactate levels in **e**. *n* = 6 mice. **f** Schematic diagram showing the experimental procedure for murine MASH models. We fed 6-week-old *Esrra*^fl/fl^ and *Esrra*^HKO^ male mice a GAN diet and injected them with CCl_4_ (0.2 µl/g of body weight) intraperitoneally twice per week for 12 weeks. **g** Hepatic TG and TC levels in **f**. *n* = 6 mice. **h** Plasma ALT and AST levels. *n* = 6 mice. **i** Gross morphology of livers, and representative images of H&E, Oil Red O, Sirius Red, F4/80 and CD11c staining of liver sections. Scale bar, 50 µm. **j**–**m** Quantification of Oil Red O- (**j**), Sirius Red- (**k**), F4/80- (**l**) and Cd11c-stained (**m**) areas were measured from liver sections in **i**. *n* = 6 mice. **n**,**o** Hepatic mRNA levels of genes involved in lipogenesis and fatty acid oxidation (**n**), fibrosis and inflammation (**o**). *n* = 6 mice. **p** Hepatic lactate levels in **f**. *n* = 6 mice. **q** Protein levels of ESRRA, PPARGC1A, Pan kla, H3K18la and H3K9la in *Esrra*^fl/fl^ and *Esrra*^HKO^ MASH livers. Results were expressed as mean ± s.d. Statistical analysis was calculated using a two-tailed Student’s *t*-test (**b**, **e**, **g**, **h**, **j** to **m** and **n** to **p**), and Wald’s test by DESeq2 (**c**). *P < 0.05, **P < 0.01 and ***P < 0.001. Schematic in **f** created in BioRender. Tongling, H. (2026) https://BioRender.com/kd0v42z.

We and others have reported that loss of ESRRA activity via genetic or pharmacological inhibition exacerbates the development of MASLD induced by rapamycin or an HFD, specifically impinging MASLD/MASH development in female mice through blockade of estrogen–ESR1 signaling^18,26^. Indeed, we confirmed the declined expression of ESRRA and PPARGC1A in female mice 8 weeks after OVX, a model of estrogen deprivation with accelerated aging features including hepatic steatosis (Supplementary Fig. 1a–c). Furthermore, ESRRA and PPARGC1A protein were also expressed at lower levels in 25-month-old aged male mice (largely equivalent to an age of 80 years in humans) accompanied with fatty liver than those in 3-month-old young male mice (Fig. 1a and Supplementary Fig. 1d,e). We further analyzed liver tissues from female mice with steatosis induced by a 28-week HFHC diet, as described in our previous study^18^ (Supplementary Fig. 1f,g). In agreement, hepatic levels of ESRRA and PPARGC1A were lower in these HFHC-fed mice than in chow diet (CD) controls (Fig. 1a). A parallel reduction was confirmed in a progressive MASH mouse model with profound liver steatosis and fibrosis induced by a well-established GAN diet combined with CCl_4_ treatment that closely replicates the features of human MASH^24^ (Fig. 1a and Supplementary Fig. 1h–j). This striking correlation of ESRRA/PPARGC1A and multiple liver injuries implies a key role of ESRRA in MASLD to MASH progression. Notably, elevated levels of lactate were observed in these injured livers (Fig. 1b and Supplementary Fig. 1k). As lactate has been recently known as a metabolite providing a lactyl donor for histone lactylation^9^, we characterized the relative levels of histone lactylation markers. Enrichment of H3K18la, H3K9la and pan-lactylation on histones was consistently observed in these models, but H3K27la levels were unchanged; however, H3K14la was only induced in the liver from the MASH model (Fig. 1a and Supplementary Fig. 1a). These findings implicate that augmentation in histone lactylation, especially H3K18la and H3K9la, due to ESRRA/PPARGC1A suppression might be a common feature during MASLD/MASH development induced by aging, estrogen deficiency, an HFHC diet and a GAN diet plus CCl_4_.

### Genetic ablation of ESRRA in hepatocytes exacerbates MASLD/MASH pathology associated with aggravated histone lactylation in mice

Next, we examined hepatic gene expression in several published transcriptome datasets (Gene Expression Omnibus (GEO)) containing human liver samples from MASLD, MASH or HCC and corresponding controls. Notably, downregulation of ESRRA was presented in patients with MASLD, MASH and HCC (Fig. 1c and Supplementary Fig. 1l). To further address whether reduced expression of hepatocyte ESRRA is a key molecular hallmark responsible for MASLD/MASH development, we used hepatocyte *Esrra*-knockout mice (AlbCre-*Esrra*^fl/fl^ mice, referred to as *Esrra*^HKO^). In line with our previous study^18^, *Esrra*^HKO^ male mice displayed mild hepatic steatosis (Fig. 1d). Importantly, hepatic loss of ESRRA led to histone lactylation together with elevated lactate levels in the liver (Fig. 1d,e). Next, we developed a MASH model in *Esrra*^HKO^ and *Esrra*^fl/fl^ male mice at the age of 18 weeks old with 12 weeks of GAN diet plus CCl_4_ administration (Fig. 1f). Although body weights and fasting blood levels of glucose and lactate were comparable in the two genotypes (Supplementary Fig. 1m–o), *Esrra*^HKO^ mice exhibited enhanced hepatic contents of TG and TC and increased blood levels of ALT and AST, indicative of liver injury (Fig. 1g,h). Analyses of liver sections in *Esrra*^HKO^ mice revealed markedly increased fat deposition, more severe fibrosis and greater macrophage infiltration than control MASH mice, specifically by H&E and Oil Red O staining for lipid droplet, Sirius Red staining for collagen deposition and immunofluorescence staining of macrophage markers F4/80 and CD11c (Fig. 1i–m). We also observed augmented hepatic glycogen contents in *Esrra*^HKO^ mice by PAS Staining (Supplementary Fig. 1p,q). Furthermore, mRNAs involved in hepatic inflammation, fibrogenesis and lipogenesis were markedly increased in *Esrra*^HKO^ mice, while the mRNAs associated with lipid oxidation were significantly declined in comparison with MASH controls (Fig. 1n,o). Importantly, *Esrra*^HKO^ MASH mice showed enhanced hepatic lactate contents and augmented expression of histone H3K18la and H3K9la compared with*Esrra*^fl/fl^ MASH mice (Fig. 1p,q). These findings demonstrate that hepatocyte ESRRA deficiency at the progressive stage of MASH propagates steatohepatitis and fibrosis accompanied with hepatic lactate accumulation and hyperlactylation on histones.

### Hepatic ESRRA is critical for mediating lactate clearance and gluconeogenesis

**Fig. 2:**
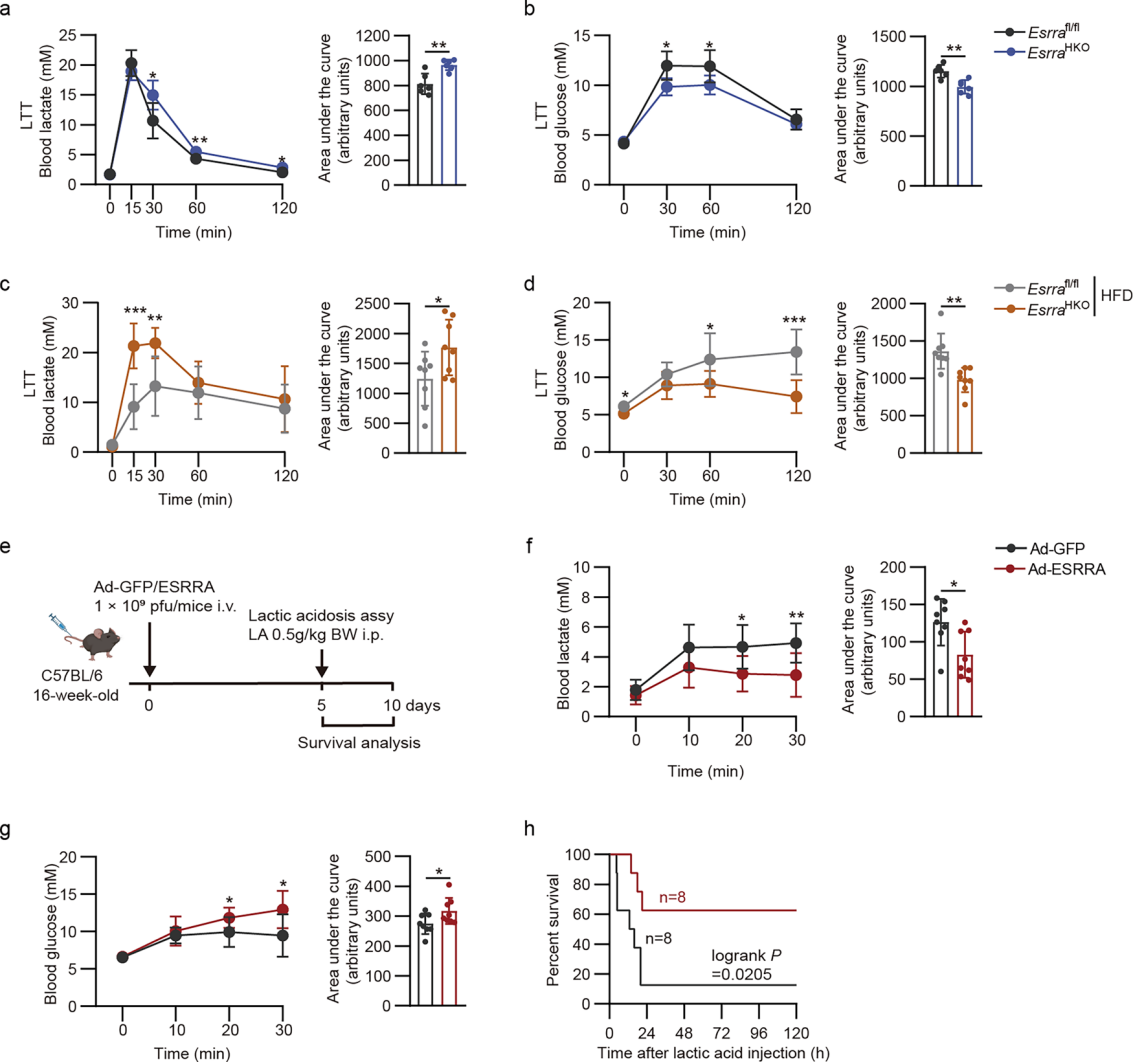
Hepatic ESRRA is required for mediating lactate clearance and gluconeogenesis in vivo. **a**,**b** An LTT was performed in 8-week-old *Esrra*^fl/fl^ and *Esrra*^HKO^ male mice with intraperitoneal injections with Lac (2 mg/g body weight). Blood lactate (**a**) and glucose (**b**) was measured serially, and the area under the curve (AUC) was calculated, respectively. *n* = 6 mice. **c**,**d** LTT comparing *Esrra*^fl/fl^ and *Esrra*^HKO^ mice after 12 weeks of HFD feeding. Blood lactate (**c**) and glucose levels (**d**) were examined serially, and the corresponding AUC was calculated. *n* = 8 mice. **e** Schematic diagram illustrating the experimental design for lactic acidosis assay. C57BL/6 male mice received intraperitoneal injections of LA (0.5 mg/g body weight) after treatment with either Ad-GFP or Ad-ESRRA for 5 days. **f**,**g** Blood lactate (**f**) and glucose (**g**) were measured at the indicated time and an AUC was also analyzed, respectively. *n* = 8 mice. **h** Survival curves were analyzed within 5 days following the challenge with LA. *n* = 8 mice. Results were expressed as mean ± s.d. Statistical analysis was performed using a two-tailed Student’s *t*-test (**a** to **d, f** and **g**) and log-rank test (**h**). *P < 0.05, **P < 0.01 and ***P < 0.001. Schematic in **e** created in BioRender. Tongling, H. (2026) https://BioRender.com/dr6ndef.

As a fundamental feature of hepatocytes, lactate can be converted to glucose robustly and quickly especially during fasting or exercise, that is, the Cori cycle^21^. To explore whether ESRRA is responsible for hepatic lactate metabolism, we performed an LTT by intraperitoneal injections of Lac in 16-h-fasted *Esrra*^HKO^ and *Esrra*^fl/fl^ control mice fed with either a normal CD or HFD for 12 weeks. Blood lactate clearance and glucose excursion were significantly decreased in *Esrra*^HKO^ mice, and greater differences between two genotypes were observed after 12-week HFD feeding (Fig. 2a–d). These findings reveal that hepatocyte ESRRA deficiency leads to compromised lactate clearance contributing to an enrichment of histone lactylation in liver.

However, L-lactic acidosis is the most common cause of metabolic acidosis and cancer acidosis^6^. As the liver is a major regulator of acid–base homeostasis besides the kidneys and lungs, patients with liver diseases are at a high risk of developing complex metabolic acid–base disorders such as severe sepsis or septic shock^27,28^. To determine whether hepatic ESRRA overexpression could overcome critical levels of hyperlactatemia in acid–base disorders, we performed lactic acidosis assays by systemic injections of a lethal dose of LA in wild-type C57BL/6 male mice infected with either an adenovirus expressing ESRRA (Ad-ESRRA) or control adenoviruses (Ad-GFP) (Fig. 2e). Compared with LA-treated control mice, the elevation of blood lactate levels was clearly attenuated in mice treated with Ad-ESRRA, accompanied by moderately higher blood glucose levels (Fig. 2f,g). Subsequently, the mice administrated Ad-ESRRA also exhibited much higher survival rates than controls (Fig. 2h). These remarkable differences demonstrate that acute ESRRA overexpression in the liver rescues mice from acidosis by metabolic engineering of lactic acid clearance. Cumulatively, these findings strongly demonstrated that hepatocyte ESRRA is a key modulator for lactate clearance, promoting the hepatic arm of the Cori cycle in pathophysiological conditions.

### Hepatocyte ESRRA directly regulates transcriptional expression of gluconeogenic enzymes LDHB and G6PC1

**Fig. 3:**
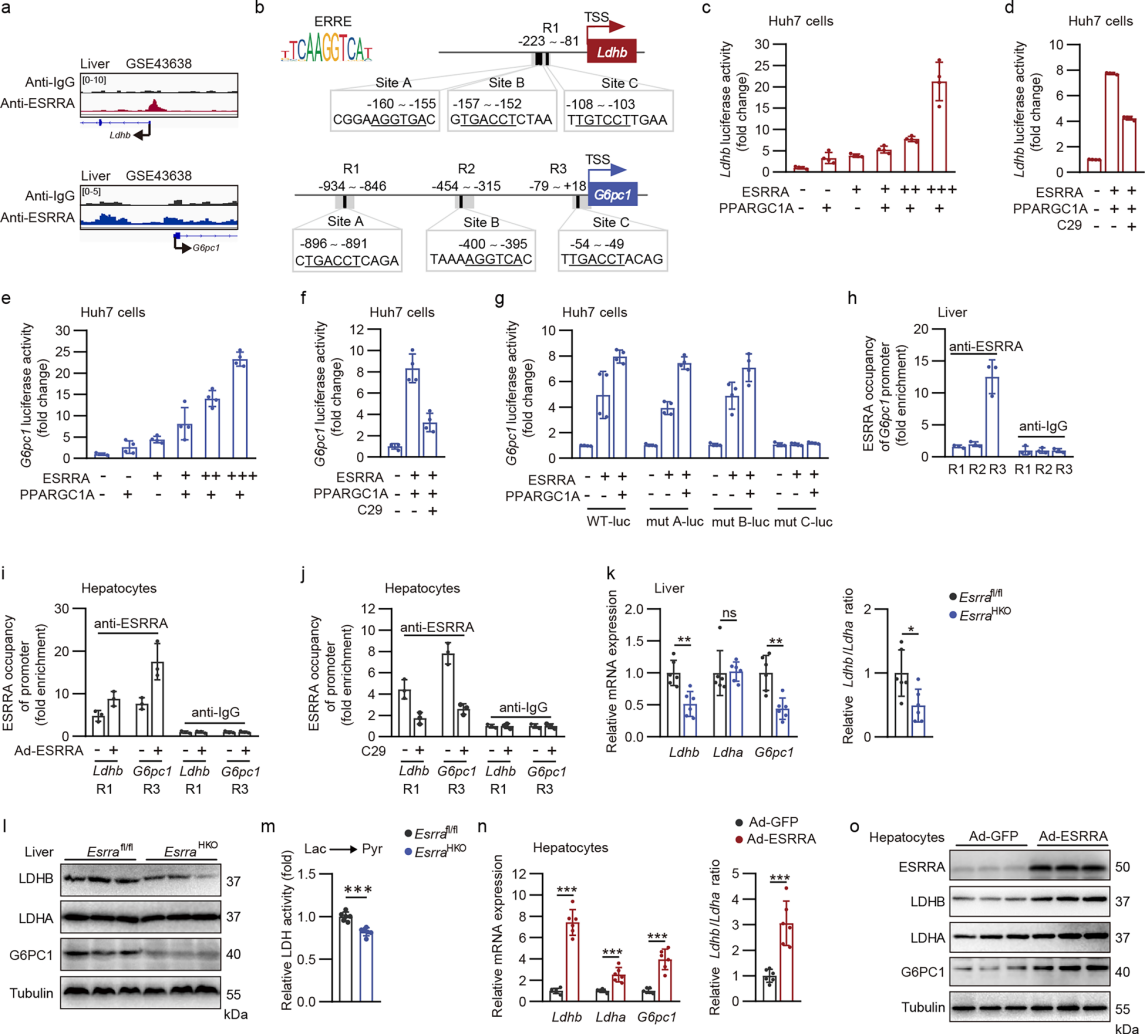
Hepatocyte ESRRA directly regulates transcriptional expression of gluconeogenic enzymes *Ldhb* and *G6pc1.* **a** Genome browser tracks of ChIP-seq signals at the ESRRA-marked *Ldhb* and *G6pc1* gene loci in mouse livers from GEO dataset GSE43638. **b** Schematic diagram displaying the potential binding sites of ESRRA on the mouse *Ldhb* and *G6pc1* promoters, with predicted ERRE sequences highlighted by underlined nucleotides. Fragments for ChIP assay shown as region 1 (R1), region 2 (R2) or region 3 (R3), respectively. **c**–**f** Luciferase reporter activities of the wild-type *Ldhb* (**c**) or *G6pc1* (**e**) promoter in Huh7 cells transfected with *Esrra*, *Ppargc1a* or control plasmids and the effects of compound 29 (C29) on the transcriptional regulation of ESRRA/PPARGC1A on the *Ldhb* promoter (**d**) or *G6pc1* promoter (**f**), respectively. *n* = 4. **g** Effects of ESRRA on indicated constructs of *G6pc1*-promoter activities were examined in Huh7 cells. *n* = 4. **h** ChIP assay showing the enrichment of ESRRA on R1, R2 and R3 of the *G6pc1* promoter in the wild-type C57BL/6 mice livers. *n* = 3 mice. **i**,**j** ChIP assay of the enrichment of ESRRA on indicated regions of *Ldhb* and *G6pc1* promoters in primary hepatocytes treated with Ad-ESRRA or Ad-GFP (**i**) and 20 μM C29 or dimethylsulfoxide (**j**), respectively. *n* = 3. **k** mRNA expression of *Ldhb*, *Ldha*, *G6pc1* and the *Ldhb*/*Ldha* ratio in the livers from *Esrra*^fl/fl^ and *Esrra*^HKO^ mice fed with a CD. *n* = 6 mice. **l** Western blot analysis of hepatic LDHB, LDHA and G6PC1 proteins from *Esrra*^fl/fl^ and *Esrra*^HKO^ mice fed with a CD. **m** The relative lactate-to-pyruvate LDH activity of mice livers as in **k**. *n* = 6 mice. **n** The mRNA levels of *Ldhb*, *Ldha*, *G6pc1* and the *Ldhb*/*Ldha* ratio in wild-type primary hepatocytes infected with Ad-ESRRA or Ad-GFP. *n* = 6. **o** Western blot analysis of indicated proteins from hepatocytes as in **n**. Results were expressed as mean ± s.d. Statistical analysis was performed using a two-tailed Student’s *t*-test (**k**, **m** and **n**). *P < 0.05, **P < 0.01 and ***P < 0.001.

It is well known that LDHB drives the conversion of lactate to pyruvate, the suppression of which contributes to augmented cellular lactate levels^29^. Notably, *Ldhb* expression can be upregulated by exercise-induced ESRRA/PPARGC1A activation in human and rodent muscle^21,22^. Nevertheless, owing to the low expression of LDHB in the liver, the regulation of LDHB expression and its function in hepatocytes has largely been overlooked for decades. However, as a rate-limiting enzyme responsible for the terminal step in the gluconeogenic pathway, G6PC1 has been implicated in the ESRRA-mediated complex transcriptional networks in liver metabolism, although its particular modulation and functional consequences have not been elucidated^30^. We then sought to delineate the direct role of ESRRA on the transcriptional regulation of these two gluconeogenic genes *Ldhb* and *G6pc1* in hepatocytes. We first examined chromatin binding of ESRRA by re-analysis of the published mouse liver ChIP sequencing (ChIP-seq) dataset (GSE43638)^26^, which revealed ESRRA occupancy at the promoter regions of both *Ldhb* and *G6pc1* (Fig. 3a). Furthermore, two previous studies have reported three ESRRA response element (ERRE) sites on the *Ldhb* promoter closely located at −155 to −156, −152 to −157 and −103 to −108 base pairs (bp) (site A, B and C) in mouse skeletal muscle^21,22^ (Fig. 3b). In addition, bioinformatics analysis suggested the presence of three putative ERREs on the *G6pc1* promoter (Fig. 3b). We then confirmed that the wild-type promoter of either *Ldhb* or *G6pc1* conferred responsiveness to ESRRA and its co-activator PPARGC1A in a dose-dependent manner, which can be suppressed by a well-studied ESRRA specific antagonist compound 29 (C29) in Huh7 cells (Fig. 3c–f). By deletion and mutation analysis of three putative ERREs on the *G6pc1* promoter, we proved that ESRRA driving the expression of *G6pc1* is dependent on site C, which was further confirmed by ChIP analysis from normal mouse liver (Fig. 3g,h). Furthermore, overexpression of ESRRA using adenovirus augmented ESRRA occupancy on each of the *Ldhb* and *G6pc1* promoters in primary hepatocytes, while ESRRA abrogation by C29 led to a decrease in this recruitment on each respective promoter (Fig. 3i,j). Consistently, either ESRRA-ablated livers or C29-treated hepatocytes resulted in declined mRNA and protein expression levels of *G6pc1 and Ldhb*, as well as the relative *Ldhb/Ldha* ratio and relative enzymatic activity of LDHB-mediated lactate-to-pyruvate conversion (Fig. 3k–m and Supplementary Fig. 2a–e). On the contrary, adenovirus-mediated ESRRA overexpression in hepatocytes led to an increase in the expression of these genes (Fig. 3n,o). Collectively, these findings elucidate that both *Ldhb* and *G6pc1* are bone fide target genes of ESRRA in hepatocytes, contributing to ESRRA-mediated lactate-derived gluconeogenesis.

### Regulation of hepatic ESRRA-mediated lactate-driven gluconeogenesis and histone lactylation by exercise is attributed to glucagon–cAMP–PKA signaling

**Fig. 4:**
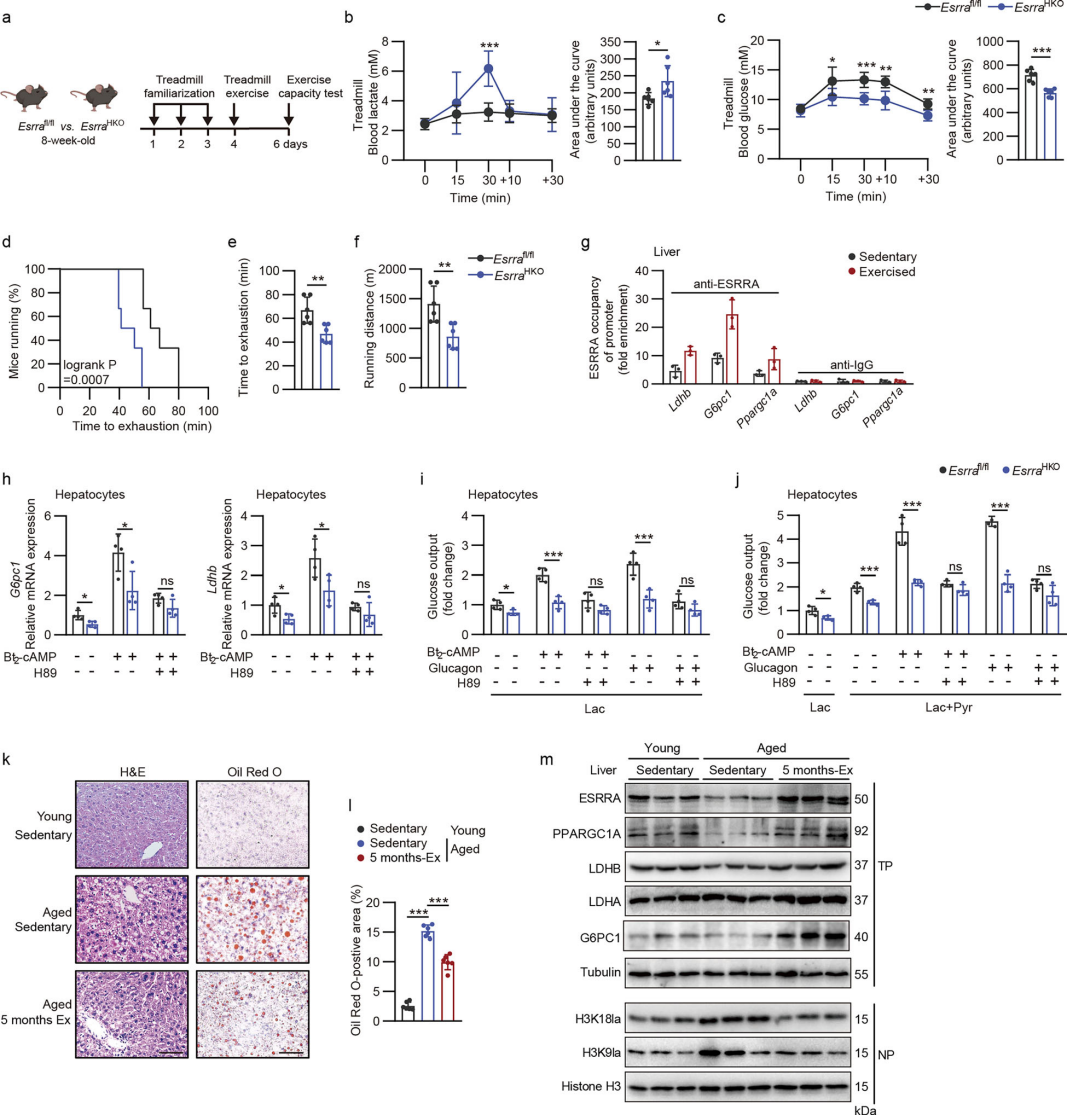
Exercise-induced ESRRA drives hepatic lactate conversion in part attributed to glucagon–cAMP–PKA signaling. **a** Schematic representation showing the experimental design for treadmill exercise and exercise capacity test in 8-week-old *Esrra*^fl/fl^ and *Esrra*^HKO^ male mice fed with a CD. **b**,**c** Blood lactate (**b**) and glucose (**c**) excursion curves were shown at the indicated time following acute treadmill exercise, and the corresponding AUC was calculated. *n* = 6 mice. **d**–**f** Endurance capacity (**d**), total time on the treadmill (**e**) and total distance ran (**f**) of maximal exercise capacity test. *n* = 6 mice. **g** ChIP assay with ESRRA antibody on *Ldhb*, *G6pc1* or *Ppargc1a* promoters in the livers from wild-type C57BL/6 mice underwent maximal endurance exercise or maintained a sedentary state. *n* = 3 mice. **h** The mRNA levels of *G6pc1* and *Ldhb* in hepatocytes pretreated for 2 h with or without 20 μM H89 and then costimulated with 100 nM glucagon or 50 μM Bt_2_-cAMP for 6 h. *n* = 4. **i**,**j** Glucose production supported by Lac (**i**) or a mixture composed of 10 mM Lac and 1 mM Pyr (Lac + Pyr) (**j**) in hepatocytes was measured after incubation with or without Bt_2_-cAMP, glucagon or H89. *n* = 4. **k** H&E and Oil Red O staining of liver sections from young mice (3 months old) and aged mice (25 months old) with or without exercise for 5 months. Scale bar, 50 µm. **l** Quantification of Oil Red O stained areas in **k**. *n* = 6 mice. **m** Western blot analysis of indicated proteins from liver tissues in **k**. Results were expressed as mean ± s.d. Statistical analysis was performed using a two-tailed Student’s *t*-test (**b**, **c**, **e**, **f** and **h** to **j**), log-rank test (**d**) and two-way ANOVA with Tukey’s post hoc test (**l**). *P < 0.05, **P < 0.01 and ***P < 0.001. Schematic in **a** created in BioRender. Tongling, H. (2026) https://BioRender.com/legpi7c.

Exercise is believed to boost liver oxidative capacity and be beneficial for liver metabolic dysfunction^31–33^. During exercise, lactate can be quickly induced from muscle cells and subsequently taken up by liver, kidney or heart for turnover and oxidation, the vast majority of which occurs in the liver for whole-body glucose homeostasis. Thus, we sought to verify whether hepatocyte ESRRA is responsible for liver lactate turnover to maintain exercise performance during acute treadmill exercise (Fig. 4a). Baseline blood levels of lactate and glucose were similar in the two genotypes; however, after 30 min of strenuous exercise, *Esrra*^HKO^ mice exhibited significantly higher blood lactate levels but lower glucose levels compared with *Esrra*^fl/fl^ mice, indicating that hepatocyte-specific ESRRA ablation reduces the conversion of lactate released from muscles during strenuous exercise (Fig. 4b,c). Time to exhaustion and running distance were markedly shorter in *Esrra*^HKO^ mice than in control mice, suggesting that defective lactate-driven gluconeogenesis caused by hepatic ESRRA deficiency compromises exercise endurance (Fig. 4d–f).

During exercise or fasting, glucagon is released into the circulation and stimulates the classic cyclic adenosine monophosphate (cAMP)–PKA signaling cascade to facilitate gluconeogenesis, and the transcriptional network of PPARGC1A is well recognized as a central mediator^5,34,35^. Hence, we postulated that ESRRA/PPARGC1A may orchestrate metabolic–epigenetic changes in response to exercise and support the benefit of exercise on the steatotic liver. We first detected that exercise as a physiological stressor rapidly enhanced transcriptional activity of ESRRA on its downstream gluconeogenic genes in the liver. As evidenced by ChIP–qPCR assays using an anti-ESRRA antibody, intense short-term exercise resulted in substantially enhanced hepatic ESRRA occupancy on each respective promoter of *Ldhb* and *G6pc1* compared with that of resting mice livers (Fig. 4g), as well as *Ppargc1a* as a positive control owing to its promoter encompassing ERRE site^15,16^. These results revealed that the physical binding of endogenous ESRRA to *Ldhb* and *G6pc1* in the liver was further promoted upon exercise induction. Furthermore, mRNA expression levels of *Ldhb* and *G6pc1* were significantly increased by dibutyl cAMP (Bt_2_-cAMP) challenge; however, ESRRA deficiency markedly eliminated such induction in primary hepatocytes (Fig. 4h). Similarly, *Ldhb* and *G6pc1* expression induced by Bt_2_-cAMP were substantially blocked in hepatocytes pretreated with PKA inhibitor H89 (Fig. 4h). These data demonstrated that hepatic ESRRA and its downstream genes *Ldhb* and *G6pc1* were tightly coupled with cAMP–PKA signaling.

We next examined the role of ESRRA on lactate-derived gluconeogenesis in primary hepatocytes by glucose output assays. Considering that lactate is the reduced form of pyruvate, either Lac was supplemented alone as the major gluconeogenic precursor or co-administered with Pyr at a ratio of 10:1 to resemble the intracellular ratio and minimize perturbation of redox state. As expected, a combined Lac + Pyr supported a higher rate of glucose production compared with that of Lac alone (Fig. 4i,j). Of note, ESRRA deletion in hepatocytes efficiently inhibited such glucose production derived from the carbon sources in both treatments, implying a notable contribution of ESRRA in mediating lactate-driven glucose production (Fig. 4i,j). Particularly, the induction of gluconeogenesis by either glucagon or Bt_2_-cAMP was significantly abrogated in *Esrra*^HKO^ primary hepatocytes, which is similar to PKA inhibition by H89, suggesting that ESRRA favors a shift from lactate to glucose in hepatocytes stimulated by classic glucagon–cAMP–PKA signaling (Fig. 4i,j).

To further confirm that the ESRRA–LDHB axis is involved in liver adaptions to chronic exercise contributing to the improvement of the age-related steatotic liver, we adopted liver tissues from mice at 25 months of age that underwent regular moderate-intensity continuous exercise training for 5 months in our previous study^25^. Compared with the age-matched sedentary group that displayed apparent MASLD features with accumulated lipid droplets in the liver sections, endurance exercise training had pronounced beneficial effects, counteracting hepatosteatosis in the aged mice (Fig. 4k,l). Importantly, chronic exercise training in aged mice substantially restored the protein expression levels of ESRRA and PPARGC1A as well as G6PC1 and LDHB, whose expression were comparable with those of young sedentary mice at 3 months of age, indicating that ESRRA–LDHB signaling played a pronounced hepatic response to exercise (Fig. 4m). In line with these findings, age-dependent histone lactylation accumulation, especially H3K18la, was significantly attenuated by endurance exercise training (Fig. 4m). Collectively, these findings suggest that hepatic ESRRA is an exercise-responsive factor together with its co-activator PPARGC1A, promoting lactate clearance via upregulation of the gluconeogenic enzyme LDHB and thus constraining histone lactylation in hepatocytes and conferring the protective effects of exercise against MASLD/MASH development.

### Exercise promotes hepatic ESRRA-mediated lactate consumption interrupting accumulated histone lactylation dependent on LDHB in the liver

**Fig. 5:**
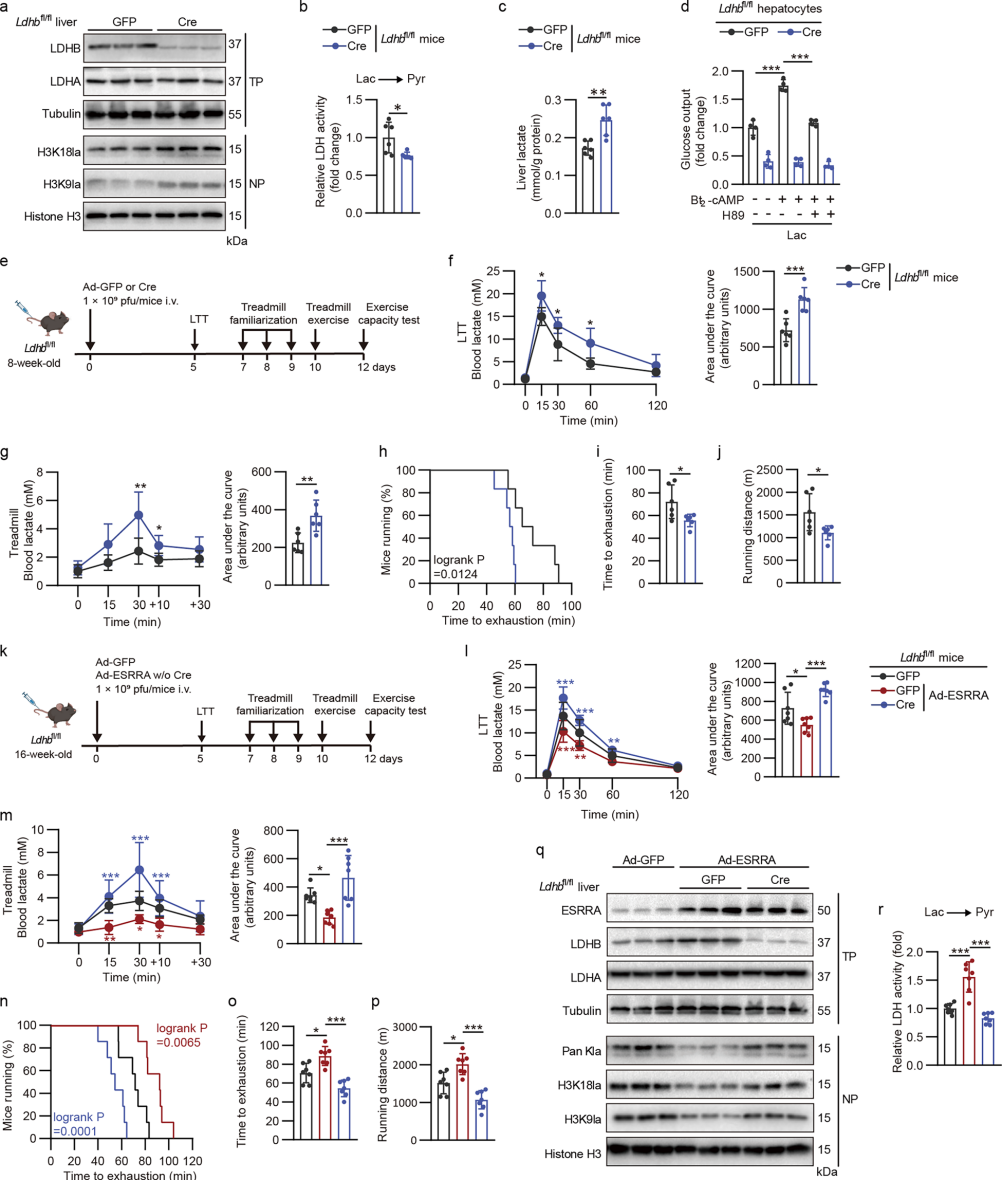
Hepatic ESRRA facilitates lactate consumption and reduces histone lactylation in an LDHB-dependent manner. **a**–**c** Protein levels of LDHB, LDHA, H3K18la and H3K9la (**a**), relative LDH activity (**b**) and lactate levels (**c**) in the livers from *Ldhb*^fl/fl^ mice treated with either Ad-Cre or Ad-GFP. *n* = 6 mice. **d** Glucose production assay supported by Lac as the substrate in *Ldhb*^fl/fl^ hepatocytes infected with Ad-Cre or Ad-GFP, pretreated for 2 h with 20 μM H89 and then costimulated with or without 50 μM Bt_2_-cAMP. *n* = 4. **e** Schematic diagram displaying the experimental procedure of LTT, treadmill exercise and Ad-Cre/GFP administration for *Ldhb*^fl/fl^ male mice. **f**,**g** Blood lactate at indicated time points and the corresponding AUC for LTT (**f**) and treadmill exercise (**g**). *n* = 6 mice. **h**–**j** Running curves (**h**), total time (**i**) and distance (**j**) to exhaustion on treadmill. *n* = 6 mice. **k** Schematic diagram showing the experimental procedure for *Ldhb*^fl/fl^ mice infected with Ad-GFP, or Ad-ESRRA with or without Ad-Cre. *n* = 7 mice. **l**,**m** Blood lactate excursion curves and the corresponding AUC for LTT (**l**) and treadmill exercise (**m**) from mice in **k**. *n* = 7 mice. **n**–**p** Running curves (**n**), time (**o**) and distance (**p**) to exhaustion on treadmill. *n* = 7 mice. **q** Western blot analysis of hepatic protein levels. **r** Relative lactate-to-pyruvate LDH activity of mice livers in **k**. *n* = 7 mice. Statistical analysis was performed using a two-tailed Student’s *t*-test (**b**, **c**, **f**, **g**, **i** and **j**), two-way ANOVA with Tukey’s post hoc test (**d**, **l**, **m**, **o**, **p** and **r**) and log-rank test (**h** and **n**). *P < 0.05, **P < 0.01 and ***P < 0.001. Schematic created in BioRender: **e**, Created in BioRender. Tongling, H. (2026) https://BioRender.com/jubpp8j; **k,** Created in BioRender. Tongling, H. (2026) https://BioRender.com/1i0zsoj.

Next, we further confirmed that hepatic levels of LDHB expression and relative activity were declined in OVX and MASH mice, associated with the reduced expression of ESRRA/PPARGC1A and G6PC1 (Fig. 1a and Supplementary Figs. 1a and 3a–c). To dissect the role of the hepatic ESRRA/LDHB–lactate axis in vivo and avoid the indirect metabolic dysfunction consequences caused by long-term deficiency of LDHB, we achieved an acute and stable liver-specific LDHB gene deletion in mice with homozygously floxed alleles encoding *Ldhb* (*Ldhb*^fl/fl^) by tail vein injections of Cre-expressing adenovirus as previously described^36^. Hepatic loss of LDHB led to augmented H3K18la and H3K9la together with elevated lactate levels in the liver without disturbance of LDHA expression, highlighting the essential role of LDHB in preserving the homeostasis of liver lactate and histone lactylation under normal conditions (Fig. 5a–c). Furthermore, *Ldhb*^fl/fl^ primary hepatocytes infected by adenoviral Cre ex vivo significantly inhibited glucose enrichment and diminished lactate-derived gluconeogenesis stimulated by Bt_2_-cAMP, suggesting that hepatocyte LDHB is required for cAMP signaling-mediated lactate turnover (Fig. 5d). Consequently, LTT and intensive treadmill exercise assays confirmed that liver LDHB deficiency reduced blood lactate clearance and compromised exercise endurance, recapitulating that of *Esrra*^HKO^ mice (Fig. 5e–j). These findings indicate that hepatocyte LDHB is an essential modulator of lactate-derived histone lactylation and required for hepatic exercise response.

To elucidate whether hepatic LDHB acts downstream of ESRRA to facilitate lactate clearance in vivo, we further examined the effects of hepatic ESRRA overexpression by tail vein administration of ESRRA-expressing adenovirus in the presence or absence of LDHB in the liver. By contrast to *Esrra*^HKO^ mice performances observed in LTT and treadmill exercise assays, hepatic ESRRA overexpression in mice accelerated blood lactate clearance, gluconeogenesis and improved maximal exercise performance (Fig. 5k–p and Supplementary Fig. 3d,e). However, these effects were impeded in liver-specific LDHB knockdown mice, indicating the roles of ESRRA on lactate turnover is dependent on LDHB (Fig. 5k–p and Supplementary Fig. 3d,e). Of note, hepatic LDHB deficiency restored the declined enrichment of H3K18la and H3K9la in the ESRRA-overexpressing liver, strongly suggesting that hepatic LDHB is essential for ESRRA-modulated histone lactylation (Fig. 5q,r). Interpreted together, these results demonstrate that the hepatic ESRRA–LDHB axis plays a critical role in lessening lactate-derived lactylation on histone lysine residues, especially under exercise conditions.

### ESRRA–LDHB signaling rewires the use of lactate as a metabolic precursor rather than a lactyl donor for histone lactylation in hepatocytes

**Fig. 6:**
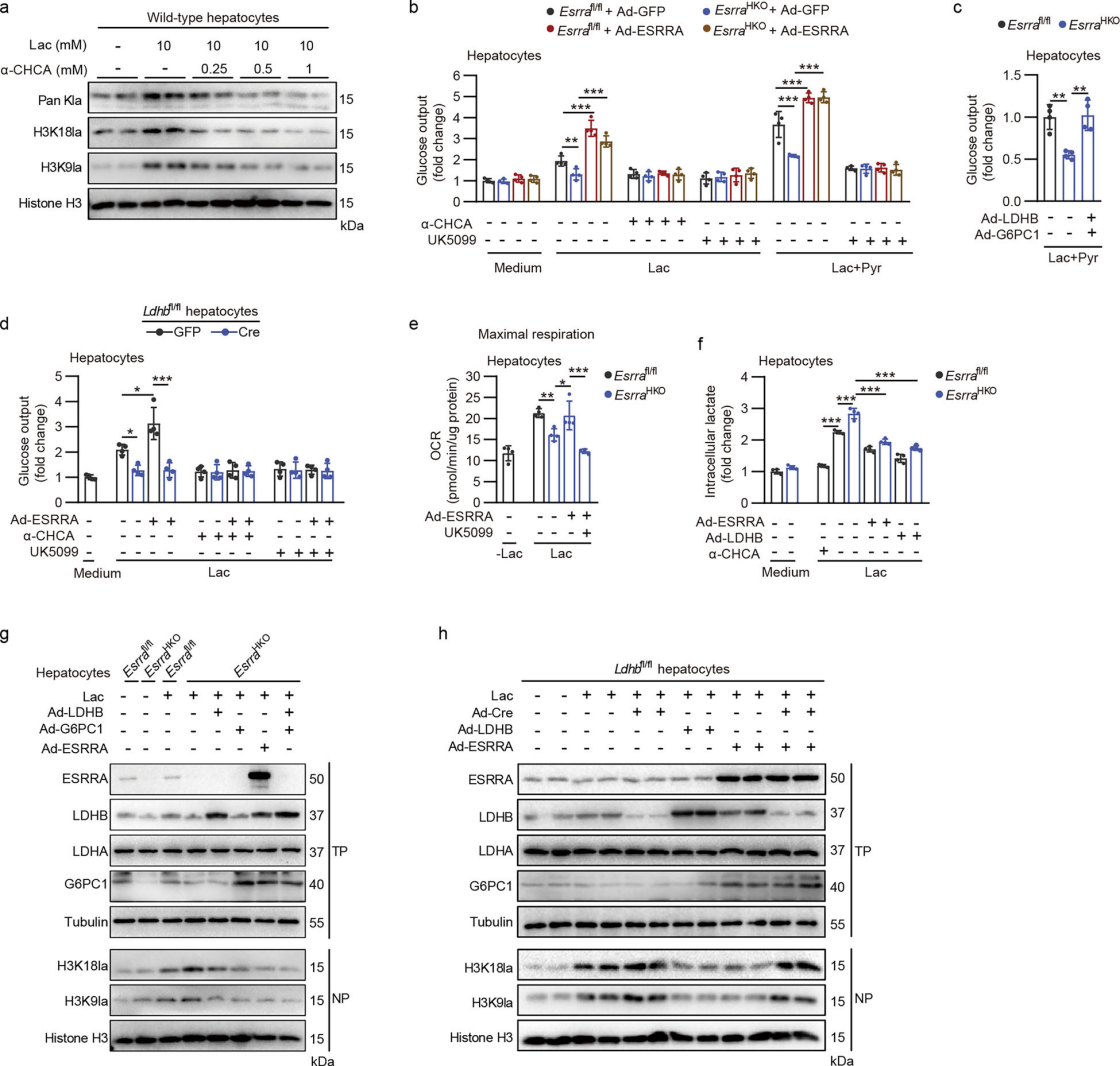
ESRRA–LDHB signaling shifts the use of lactate from a lactyl donor for histone lactylation to a metabolic precursor in hepatocytes. **a** Protein levels of Pan kla, H3K18la and H3K9la in hepatocytes treated with different doses of the MCT inhibitor α-CHCA for 24 h in the presence of 10 mM Lac. **b** Glucose production in hepatocytes extracted from *Esrra*^fl/fl^ and *Esrra*^HKO^ mice, supported by Lac with or without Pry, was measured after incubation with vehicle, α-CHCA or the MPC inhibitor UK5099 for 6 h. *n* = 4. **c** Gluconeogenesis by primary hepatocytes isolated from *Esrra*^fl/fl^ and *Esrra*^HKO^ mice, supported by Lac + Pyr. Hepatocytes were infected with Ad-LDHB and Ad-G6PC1 or with Ad-GFP. *n* = 4. **d** Glucose production supported by Lac in hepatocytes extracted from *Ldhb*^fl/fl^ mice treated with Ad-GFP or Ad-Cre, with or without Ad-ESRRA, α-CHCA or UK5099. *n* = 4. **e** Evaluation of OCR showing maximal respiration of mitochondria in hepatocytes after acute exposure to lactate. *Esrra*^fl/fl^ and *Esrra*^HKO^ hepatocytes were infected with Ad-ESRRA or Ad-GFP and then treated with UK5099 or vehicle for 6 h. *n* = 4. **f** Intracellular lactate levels in *Esrra*^fl/fl^ and *Esrra*^HKO^ hepatocytes with indicated treatments. *n* = 4. **g** Western blot analysis of ESRRA, LDHB, LDHA, G6PC1, H3K18la and H3K9la in hepatocytes from *Esrra*^fl/fl^ and *Esrra*^HKO^ mice with the indicated treatments in the presence of 10 mM lactate. **h** Western blot analysis of the indicated proteins in *Ldhb*^fl/fl^ hepatocytes infected with Ad-Cre or Ad-GFP as well as Ad-LDHB or Ad-ESRRA in the presence of 10 mM lactate. Statistical analysis was performed using two-way ANOVA with Tukey’s post hoc test (**b**–**f**). *P < 0.05, **P < 0.01 and ***P < 0.001.

Circulating lactate can directly enter into the TCA cycle as a primary source of carbon in all tissues as evidenced by ^13^C-lactate extensively labeling TCA-cycle intermediates^37–39^. Given that ESRRA is a master regulator of mitochondrial biogenesis and oxidative phosphorylation (OXPHOS) in response to metabolic stress including fasting, calorie restriction and exercise, we next defined the ESRRA/LDHB-directed carbon flow in isolated primary hepatocytes. We first verified that histone lactylation accumulations, including pan-K, H3K18 and H3K9, can be induced by exogenous 10 mM Lac treatment in wild-type hepatocytes (Fig. 6a). However, α-cyano-4-hydroxycinnamic acid (α-CHCA), an inhibitor of monocarboxylate transporters (MCTs) that is responsible for proton–lactate symport, dose-dependently inhibited this lactylation accumulation (Fig. 6a). These findings indicated that extrahepatic lactate directly shuttles into hepatocytes, acting as a substrate for histone lactylation in hepatocytes. Furthermore, in primary hepatocytes isolated from fasted *Esrra*^HKO^ and *Esrra*^fl/fl^ mice, glucose production from the precursors lactate or Lac + Pyr was dramatically inhibited in ESRRA-ablated hepatocytes, which was rescued by ESRRA overexpression, suggestive of a reverse gluconeogenic flow involved in histone lactylation modifications but not glycolysis (Fig. 6b). By contrast, treatments by α-CHCA or UK5099, an inhibitor targeting mitochondrial pyruvate carrier (MPC), robustly abolished ESRRA-mediated gluconeogenesis (Fig. 6b). Consistently, C29 treatment recapitulated similar results in hepatocytes (Supplementary Fig. 4a). Similarly, a combination of LDHB and G6PC1 overexpression also restored glucose production derived from Lac + Pyr in hepatocytes from *Esrra*^HKO^ mice (Fig. 6c). Importantly, ESRRA overexpression cannot rescue lactate-derived glucose production in LDHB-abrogated hepatocytes, confirming that LDHB is required for ESRRA-mediated gluconeogenesis in hepatocytes (Fig. 6d). These results delineate that MCT- and MPC-driven carbon transportation including mitochondrial pyruvate shuttling enables ESRRA–LDHB–G6PC1 axis-directed carbon flow in hepatocytes.

We next sought to determine whether ESRRA defect in hepatocytes impacts bioenergetics when supplying lactate as a fuel. We measured the mitochondrial OCR in *Esrra*^HKO^ and *Esrra*^fl/fl^ hepatocytes. Control hepatocytes challenged with lactate as a substrate exhibited heightened states of maximal OCR compared with those without lactate treatment, confirming that lactate can be an alternative fuel in hepatocytes in the absence of glucose or other carbon sources (Fig. 6e and Supplementary Fig. 4b). However, *Esrra*^HKO^ hepatocytes displayed reduced maximal OCR that was reversed by ESRRA reintroduction (Fig. 6e and Supplementary Fig. 4b). By contrast, cotreatment with UK5099 blocked maximal OCR fueled by lactate, indicating that such mitochondrial respiration arose from pyruvate that is oxidized from lactate (Fig. 6e and Supplementary Fig. 4b). As expected, ESRRA overexpression in hepatocytes indeed increased the mRNA levels of TCA-cycle and OXPHOS-associated genes as well as MPC1 (Supplementary Fig. 4c), all of which were potential target genes of ESRRA as previously reported^30,40^. On the contrary, the mRNA expression of these genes was reduced in the liver of *Esrra*^HKO^ mice (Supplementary Fig. 4d). These findings imply that the boosted mitochondrial respiration enabled by ESRRA might further enhance the oxidative disposal of lactate in hepatocytes following ESRRA/LDHB-triggered lactate conversion to pyruvate.

As exogenous lactate can be either a lactyl donor for histone lactylation or provide carbon sources for ESRRA-mediated catabolism in hepatocytes, we next queried whether ESRRA rewires the use of lactate away from being a signaling molecule in primary hepatocytes. We first confirmed that intracellular lactate levels varied in hepatocytes correlated with lactate transport and clearance rates, as shown through α-CHCA diminishing exogenous lactate-induced intracellular lactate accumulation, while ESRRA ablation led to an increased level that could be constrained by enforced expression of either ESRRA or LDHB (Fig. 6f). Concomitant with intracellular lactate accumulation, lack of ESRRA resulted in elevated levels of H3K18la and H3K9la, which was further exaggerated in hepatocytes supplemented with exogenous lactate (Fig. 6g). On the contrary, overexpression of ESRRA, LDHB and G6PC1, respectively, led to reduced levels of H3K18la and H3K9la in hepatocytes from *Esrra*^HKO^ mice (Fig. 6g). We further verified that LDHB-ablation also accumulated H3K18la and H3K9la and restrained the effects of ESRRA overexpression in hepatocytes (Fig. 6h). These results clarify that ESRRA reduces enrichment of histone lactylation by shifting lactate toward consumption through LDHB. Taken together, these findings suggest that hepatic ESRRA facilitates lactate consumption as a metabolic precursor but restrains it as a signaling molecule for epigenetic modification.

### AAV-mediated restoration of hepatic ESRRA reduces histone lactylation and ameliorates MASLD/MASH

**Fig. 7:**
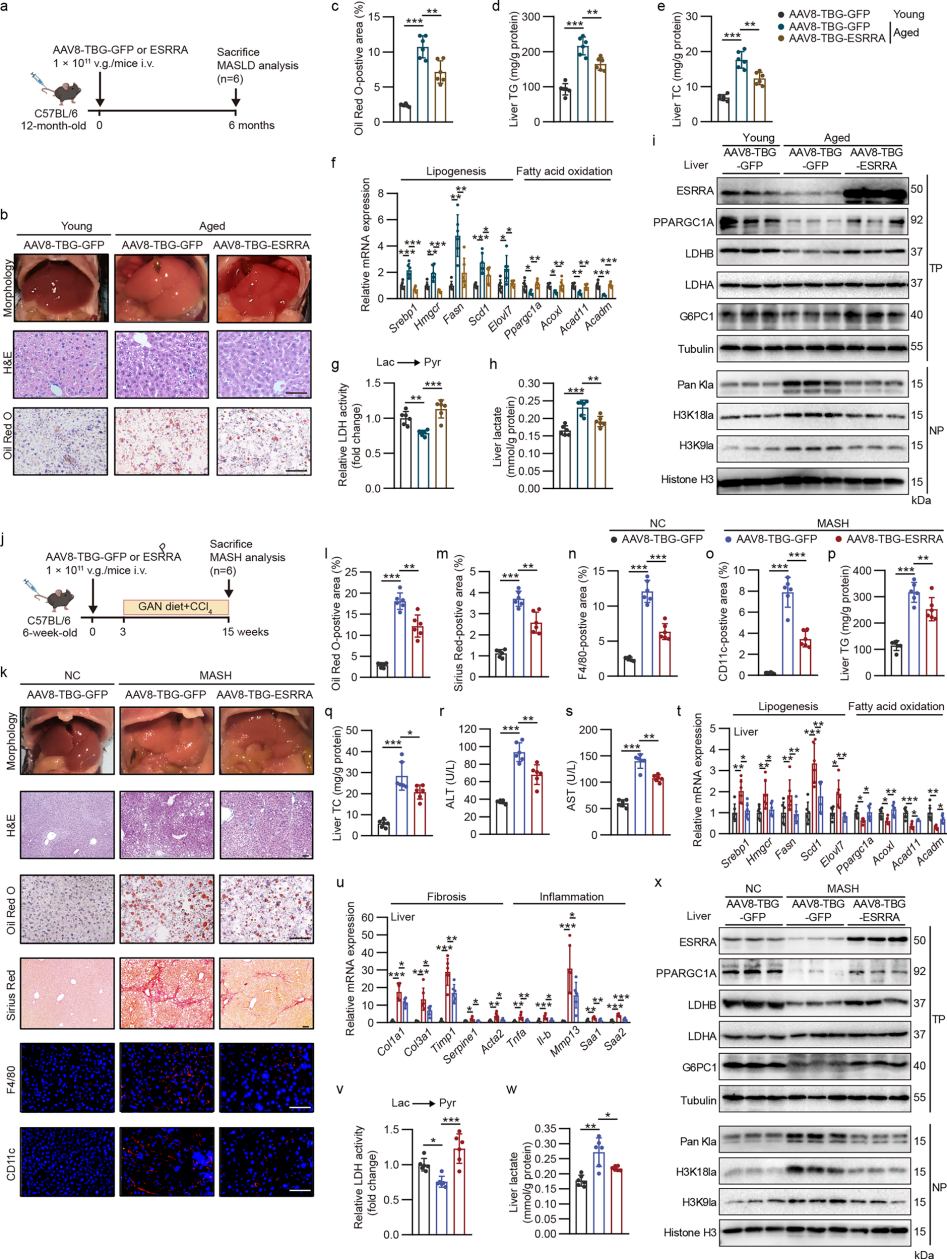
Hepatocyte-specific restoration of ESRRA expression reduces histone lactylation and ameliorates liver steatosis, fibrosis and inflammation in murine MASLD/MASH model. **a** Schematic overview of the experimental design for age-related MASLD model with the treatment of AAV8-TBG-ESRRA or AAV8-TBG-GFP. *n* = 6 mice. **b** Representative liver morphology and histological staining, including H&E and Oil Red O. Scale bar, 50 µm. **c** Quantification of Oil Red O in **b**. *n* = 6 mice. **d**,**e** Hepatic TG (**d**) and TC (**e**) levels. *n* = 6 mice. **f** Hepatic mRNA expression of genes involved in lipogenesis and fatty acid oxidation. *n* = 6 mice. **g**,**h** Relative lactate-to-pyruvate LDH activity (**g**) and lactate levels (**h**) in livers. *n* = 6 mice. **i** Western blot analysis of the indicated hepatic proteins. **j** Schematic diagram illustrating the experimental design for the MASH model with the treatment of AAV8-TBG-ESRRA or AAV8-TBG-GFP. *n* = 6 mice. **k** Liver morphology and H&E, Oil Red O, Sirius Red, F4/80 and CD11c staining in liver sections. Scale bar, 50 µm. **l**–**o** Quantification of Oil Red O- (**l**), Sirius Red- (**m**), F4/80- (**n**) and CD11c-stained (**o**) areas in **k**. *n* = 6 mice. **p**,**q** Hepatic TG (**p**) and TC (**q**) levels. *n* = 6 mice. **r**,**s** Plasma levels of ALT (**r**) and AST (**s**). *n* = 6 mice. **t**,**u** Hepatic mRNA levels of genes participating in lipogenesis and fatty acid oxidation (**t**) and fibrosis and inflammation (**u**). *n* = 6 mice. **v**,**w** Relative lactate-to-pyruvate LDH activity (**v**) and lactate levels (**w**) in the livers from mice in **j**. *n* = 6 mice. **x** Western blot analysis showing the indicated hepatic proteins. Results were expressed as mean ± s.d. Statistical analysis was performed using two-way ANOVA with Tukey’s post hoc test (**c** to **h** and **l** to **w**). *P < 0.05, **P < 0.01 and ***P < 0.001. Schematic created in BioRender: **a,** Created in BioRender. Tongling, H. (2026) https://BioRender.com/6kwvtww; **j**, Created in BioRender. Tongling, H. (2026) https://BioRender.com/1gpipxf.

Given that defective epigenetic modifications might be a common mechanism behind chronic liver disease pathology underlying different risk factors, we speculated that ESRRA is a key modulator linking hepatocellular metabolism and histone lactylation to protect against liver injury. To determine therapeutic tractability of hepatocyte ESRRA in MASLD/MASH progression, we used liver-targeted AAV8-expressing ESRRA or GFP that contains a hepatocyte-specific TBG promoter (AAV8-TBG-ESRRA or -GFP), which were delivered via tail vein injection to establish stable expression in mice (Fig. 7a). In an age-related MASLD model, wild-type male mice received AAVs at 12 months of age and were analyzed at 18 months (Fig. 7a). As expected, aged mice exhibited increased body weight as well as MASLD-associated features, while fasting plasma glucose levels remained unchanged (Supplementary Fig. 5a–d). Importantly, AAV8-TBG-ESRRA markedly mitigated MASLD in aged mice, as evidenced by reduced hepatic steatosis and lower serum ALT and AST levels (Fig. 7b–e and Supplementary Fig. 5c,d). Furthermore, ESRRA overexpression decreased the mRNA levels of lipogenic and inflammatory genes while increasing the expression of fatty acid oxidation genes, supporting a protective role against age-induced hepatic steatosis (Fig. 7f and Supplementary Fig. 5e). Restored ESRRA expression also rescued hepatic levels of LDHB, G6PC and PPARGC1A in aged mice, accompanied by reduced hepatic LDH activity and lactate levels (Fig. 7g–i and Supplementary Fig. 5f). Importantly, AAV8-TBG-ESRRA treatment greatly lowered levels of H3K18la, H3K9la and pan-lactylation (Fig. 7i). These results indicate that hepatocyte-specific restoration of ESRRA effectively ameliorates age-related MASLD, which is associated with reduced histone lactylation.

To further evaluate the efficacy of ESRRA in attenuating liver fibrosis, we administrated AAV8-TBG-ESRRA or AAV8-TBG-GFP to MASH mice subjected to 12 weeks of GAN diet plus CCl_4_ treatment (MASH-ESRRA/GFP mice) (Fig. 7j). All three groups exhibited no differences in body weights and fasting plasma levels of glucose and lactate (Supplementary Fig. 5g–i). As expected, compared with AAV-TBG-GFP-treated normal controls (NC), MASH-GFP mice exhibited more severe liver steatosis, fibrosis and inflammation (Fig. 7k–s). However, AAV8-TBG-ESRRA overexpression in hepatocytes substantially ameliorated these MASH features, as evidenced by reduced hepatic concentrations of TG and TC, declined blood ALT and AST levels, decreased lipid retention, glycogen contents and collagen deposition and fewer infiltrations of F4/80 or CD11c-positive macrophages by histological analysis in the liver sections (Fig. 7k–s and Supplementary Fig. 5j,k). Moreover, the mRNA levels of genes involved in inflammation, fibrogenesis and lipogenesis were markedly repressed in MASH-ESRRA mice livers but those of lipid oxidation-related genes were higher than the corresponding controls (Fig. 7t,u). Consistently, the expression of ESRRA/PPARGC1A as well as LDHB and G6PC was lost during MASH development but LDHA remained unchanged (Fig. 7x and Supplementary Fig. 5l). On the contrary, hepatocyte-specific ESRRA restoration in the MASH mice rescued hepatic expression of its targets genes *LDHB*, *G6PC* and *PPARGC1A*, as well as rectified relative LDH activity and liver lactate levels. (Fig. 7v–x and Supplementary Fig. 5l). Importantly, we observed that forced ESRRA expression profoundly alleviated the accumulation of H3K18la, H3K9la and pan-lactylation in MASH livers, indicating that long-term overexpression of ESRRA in hepatocytes by AAV8-TBG-ESRRA efficiently abolished lactate-derived histone lactylation in vivo (Fig. 7x). These results strongly suggest that hepatocyte ESRRA plays a beneficial role to prevent MASH progression, which is correlated with reduced histone lactylation.

### Hepatic ESRRA represses H3K18la-marked genes that are correlated with MASLD/MASH progression

**Fig. 8:**
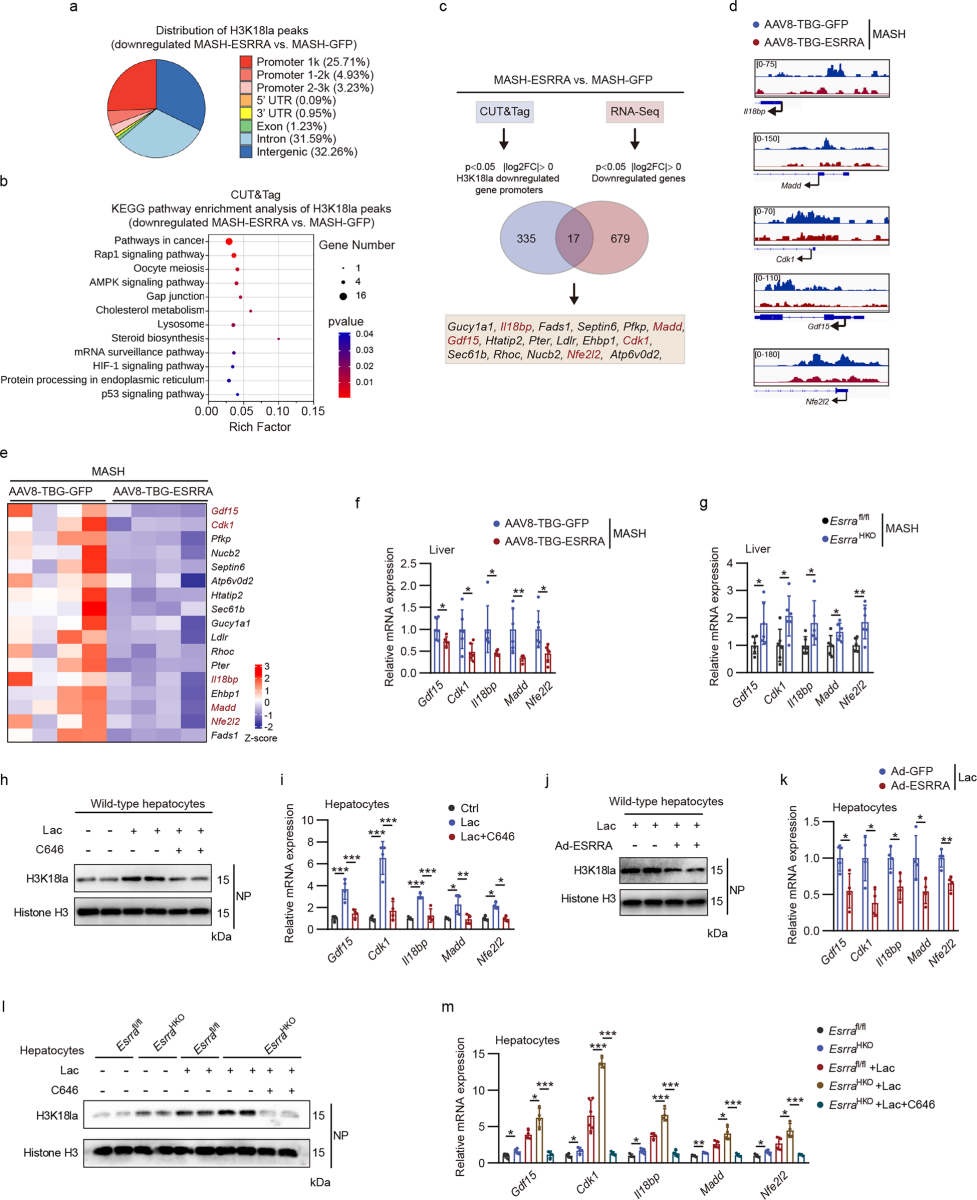
Transcriptional consequences of ESRRA and H3K18la involved in MASLD/MASH progression. **a** Genome-wide distribution of downregulated H3K18la-binding peaks at annotated genomic regions in MASH-ESRRA mouse liver compared with MASH-GFP control by CUT&Tag analysis. **b** KEGG analysis of the downregulated H3K18la binding peaks at candidate target genes in MASH-ESRRA mouse liver. **c** Venn diagram depicting 17 downregulated candidate genes with decreased H3K18la modification by integrative analysis of CUT&Tag and RNA-seq data. **d** Genome browser tracks of CUT&Tag signal at the H3K18la-marked *Il18bp, Madd*, *Gdf15*, *Cdk1* and *Nfe2l2* gene loci. **e** Heat map depicting 17 candidate genes in **c** from RNA-seq analysis. **f** Hepatic mRNA levels of H3K18la-marked genes in MASH mice treated with AAV8-TBG-GFP or AAV8-TBG-ESRRA. *n* = 6 mice. **g** Hepatic mRNA expression of H3K18la-marked genes in *Esrra*^fl/fl^ and *Esrra*^HKO^ MASH mice. *n* = 6 mice. **h**,**i** H3K18la levels (**h**) and mRNA expression of H3K18la-marked genes (**i**) in wild-type primary hepatocytes cultured with 10 mM lactate alone or with C646 treatment (100 μM). **j**,**k** Analysis of H3K18la levels (**j**) and mRNA expression of H3K18la-marked genes (**k**) in hepatocytes treated with 10 mM lactate after infection with Ad-ESRRA or Ad-GFP. **l**,**m** H3K18la levels (**l**) and mRNA expression of H3K18la-marked genes (**m**) in *Esrra*^fl/fl^ and *Esrra*^HKO^ hepatocytes after treatment with vehicle, 10 mM lactate or 10 mM lactate combined with 100 μM C646. Results were expressed as mean ± s.d. Statistical analysis was performed using a two-tailed Student’s *t*-test (**f**, **g** and **k**) and two-way ANOVA with Tukey’s post hoc test (**i** and **m**). *P < 0.05, **P < 0.01 and ***P < 0.001.

To elucidate the functional significance of ESRRA-dependent histone lactylation on transcriptional regulation, particularly H3K18la, we first performed genome-wide CUT&Tag followed by high-throughput DNA sequencing using anti-H3K18la antibodies in MASH-ESRRA mice liver tissues and MASH-GFP controls. Analysis of the genome-wide distribution of H3K18la showed that hepatic ESRRA overexpression resulted in a reduction of H3K18la chromatin occupancy at transcription start sites, as indicated by 1054 downregulated H3K18la binding peaks, of which 33.87% were distributed within promoter regions (≤3 kb) (Fig. 8a). Next, we conducted bulk RNA sequencing (RNA-seq) analysis and observed 696 genes that were expressed significantly fewer in MASH-ESRRA livers than in controls by differentially expressed gene (DEG) analysis (log_2_FC >0 (where FC is fold change), *P* < 0.05) (Supplementary Fig. 6a,b). Next, we performed KEGG and GO Biological Process pathways analysis for the 352 downregulated genes targeted by H3K18la and 696 downregulated genes in liver tissues with ESRRA overexpression (Fig. 8b and Supplementary Fig. 6c–e). Multiple pathways involved in carcinogenic and metabolic mechanisms were enriched as illustrated by KEGG enrichment analysis, such as pathways in cancer, HCC, cellular senescence, p53 signaling and Rap1 signaling as well as cholesterol metabolism, steroid biosynthesis, AMPK signaling and FoxO signaling (Fig. 8b and Supplementary Fig. 6c). The GO Biological Process analysis revealed that downregulated H3K18la-marked genes were involved in the regulation of protein metabolic process and endoplasmic reticulum stress, whereas downregulated transcripts encompassed multiple genes that are associated with cell cycle and cell division in MASH-ESRRA livers (Supplementary Fig. 6d,e). These data suggest that ESRRA-modulated H3K18la repression may reduce the transcription of key genes encoding known metabolism and tumorigenesis.

To further identify H3K18la-specific genes regulated by ESRRA, we combined CUT&Tag with RNA-seq data and identified 17 candidate genes in MASH-ESRRA livers with both low H3K18la occupancy at the promoter region and reduced mRNA expression (Fig. 8c–e and Supplementary Fig. 6f). Among these candidate genes, *Il18bp*, *Madd*, *Gdf15, Cdk1* and *Nfe212* have been reported to be involved in immune regulation, fibrosis and tumorigenesis^41–45^. Importantly, by informatics analysis of several published GEO datasets, we confirmed the transcriptome levels of these genes were increased in patients with liver steatosis, fibrosis or tumor compared with the respectively controls (Supplementary Fig. 6g–k). Subsequently, we found that hepatic ESRRA overexpression in aged MASLD mice significantly reduced the mRNA expression of these H3K18la-marked genes (Supplementary Fig. 6l). Moreover, we verified mRNA levels of these genes in liver tissues from either hepatic ESRRA-overexpression or -knockout mice in comparison with their MASH controls, respectively, whose expression were positively associated with MASH severity but negatively correlated with ESRRA expression (Fig. 8f,g and Supplementary Fig. 6m,n). Together, these findings imply that ESRRA-mediated suppression of these H3K18la-marked genes plays a key role in mitigating MASLD/MASH progression.

Acetyltransferase p300 has been reported to catalyze histone lactylation using lactyl-CoA as a substrate, and the p300 inhibitor C646 can reduce H3K18la levels^46,47^. We next assessed whether lactate-driven H3K18la directly regulates these disease-associated genes. Treating wild‑type hepatocytes with exogenous lactate increased both H3K18la levels and the mRNA expression of H3K18la‑marked genes, *Gdf15*, *Cdk1*, *Il18bp*, *Madd* and *Nfe2l2* (Fig. 8h,i). Conversely, p300 inhibitor C646 attenuated lactate-driven H3K18la and correspondingly decreased the mRNA levels of these genes, confirming their transcriptional regulation by H3K18la (Fig. 8h,i). Notably, enforced ESRRA overexpression recapitulated the effect of C646, whereas ESRRA deficiency resulted in the opposite phenotype (Fig. 8j–m). Similar to its effect in wild-type hepatocytes, C646 treatment blocked the induction of H3K18la target genes in ESRRA-deficient hepatocytes, even in the presence of lactate supplementation (Fig. 8l,m). Collectively, these results indicate that hepatocyte ESRRA modulates *Gdf15*, *Cdk1*, *Il18bp*, *Madd* and *Nfe2l2* by reducing lactate-driven H3K18la.

## Discussion

Elevated lactate or lactic acid in circulation or tissues contributes to various diseases, the mechanisms of which are attributed to multifactorial roles of lactate in energy metabolism, signal transduction and posttranslational modification^6,7,9^. Intra-organ and interorgan lactate shuttles are subverted between driver (producer) and recipient (consumer) cells in metabolic circuits, in which normal hepatocytes act as recipient cells with high gluconeogenic capacity of lactate disposal. In the present study, we observe that ESRRA/PPARGC1A expression is declined in pathological liver samples of MASLD/MASH, constituting a dynamic interplay between metabolic and epigenetic pathways in regulating hepatocyte function. We characterize that hepatocyte ESRRA responding to exercise regulates hepatic lactate clearance, which requires LDHB. AAV-mediated restoration of hepatic ESRRA alleviates MASH development, rectifying accumulated lactate-driven H3K18la that acts as a transcription initiator of key genes regulating liver pathogenesis. Mechanistically, we delineate that ESRRA modulates the transcriptional expression of *Ldhb* and *G6pc1* in hepatocytes, which can be enhanced by exercise in part owing to glucagon–cAMP–PKA signaling. In this way, ESRRA rewires lactate for consumption rather than histone lactylation, underscoring a bridging role of ESRRA in gluconeogenic and epigenetic crosstalk in hepatocytes.

Emerging evidence shows that the novel histone Kla modification driven by glycolytic–epigenetic coordination directly regulates gene expression involved in various pathophysiological processes. For example, accumulated lactate in the tumor microenvironment (TME) potently induces H3K18 lactylation that upregulates METTL3 and therefore RNA *N*^6^-methyladenosine modification in tumor-infiltrating myeloid cells, promoting the immunosuppressive capacity of myeloid cells and constraining the efficacy of clinical immunotherapy^47^. Elevated H3K18la levels are also found to potentiate the immune escape of non-small-cell lung cancer cells^48^. Similarly, elevated lactylation levels of mitochondrial fission 1 protein and H3K18 are found in patients with sepsis who produce high levels of blood lactic acid, exacerbating sepsis-induced acute kidney injury^11,12^. Notably, hexokinase 2-induced lactate from glycolysis during HSC activation facilitates lactylation on H3K18la, which further stimulates HSC activation-associated genes and promotes CCl_4_-induced liver fibrosis in mice^10^. Furthermore, TME resident fibroblasts use extracellular lactate for collagen production, facilitating tumor desmoplasia^7^. We noticed that elevated hepatic lactate levels are accompanied by enrichment of pan-histone lactylation, especially H3K9la and H3K18la, in steatotic or fibrotic livers caused by distinct risk factors including aging, estrogen deficiency and a GAN diet together with CCl_4_ challenging, suggestive of an imbalance between lactate production and consumption. Thus, it is plausible that an increase in localized or circulating lactate caused by high glycolysis or insufficient consumption might exaggerate the progression of liver pathology. Indeed, recent studies have found that three key rate-limiting gluconeogenic enzymes, G6PC1, PCK1 and fructose 1,6-bisphosphatase 1 (FBP1), are downregulated in human liver tumors, implicating an involvement of an abnormal gluconeogenic pathway in liver cancer initiation or progression^49–52^.

Exercise is a well-known physiological stressor to rapidly alter whole-body energy and glucose homeostasis, driving metabolic benefits against fatty liver^31^. During exercise, euglycemia is maintained via hepatic glycogenolysis and an increased contribution from gluconeogenesis; meanwhile, glucagon released from the pancreas stimulates hepatic uptake of gluconeogenic precursors such as muscle glycolysis-derived lactate to maintain hepatic glucose output (referred to as the Cori cycle)^5^. As a well-known fasting hormone, glucagon is involved in a classic glucagon–cAMP–PKA signaling in response to exercise or starvation. Given the great interest in glucagon/GLP-1/GIP agonists for the treatment of MASL/MASLD/MASH, a recent clinical study performed in patients with fatty liver reveals that an acute physiological increase in plasma glucagon concentrations augments in vivo rates of hepatic mitochondrial oxidation associated with an increase in glucose production in patients with MASLD, suggesting a possibility that chronic hyperglucagonemia leads to reductions in hepatic steatosis^53^. Another similar study in rodents also shows that chronic glucagon treatment-reversing hepatic steatosis is achieved by increasing mitochondrial fat oxidation^54^. Furthermore, exercise can promote mitochondrial biogenesis and fatty acid oxidation in hepatocytes to attenuate hepatosteatosis via the cAMP–PKA–CREB signaling pathway^55^. As a well-established transcriptional factor responding to starvation, ESRRA has been reported to be regulated by cAMP signaling in either hepatocytes or extrahepatic cells^56–58^. In accordance with previous studies^18,26^, we found that enforced ESRRA overexpression improves fatty acid oxidation-associated gene expression, impeding hepatic lipid accumulation in MASH mice. Notably, exercise training can induce considerable expression of ESRRA, PPARGC1A and LDHB in skeletal muscle^22,59–61^. In the current study, we further demonstrate that ESRRA/PPARGC1A and the downstream genes *LDHB* and *G6PC1* can be stimulated by glucagon–cAMP–PKA signaling, which facilities lactate-derived gluconeogenesis in hepatocytes. In line with our observation, a recent study reveals that hepatic androgen receptor mediates glucagon activity in orchestrating glucose and lipid metabolism, which is depend on ESRRA–PPARGC1A signaling^62^. In this way, an ESRRA-mediated decline in lactate accumulation might serve as an overarching signal for fine-tuning lipid catabolic processes and H3K18la modification tied to gluconeogenesis during exercise.

Endurance training leads to functional improvements in hepatic mitochondria, which may play a critical role in oxidizing not only lactate but also fat to provide ATP and substrates to fuel the TCA cycle and gluconeogenesis. On the contrary, in primary hepatocytes, accumulated intracellular lactate results in defective mitochondrial bioenergetics contributing to organ failure in a septic shock mouse model of lactic acidosis^8^. A recent study also reports that hepatocyte PPARGC1A expression is compromised in acetaminophen-induced acute liver injury accompanied by suppressed LDHB expression, mitochondrial lactate accumulation and protein lactylation, leading to mitochondrial dysfunction^63^. It is worth noting that liver ESRRA overexpression causes resistance to lactic acidosis and improves survival rate by enhancing hepatic lactate clearance, suggesting a protective role of ESRRA on mitochondrial dysfunction due to boosted mitochondrial respiratory capacity and lactate shuttling to recipient hepatocytes. By OCR assay in hepatocytes, ESRRA overexpression results in an increase in mitochondrial oxidative activity indicating increased channeling of lactate/pyruvate to the TCA cycle for energy production, which is confirmed by the augmented mRNA expression of a cluster of genes involved in these mitochondrial pathways. It will be of interest to define the lactylation of hepatocellular mitochondrial proteins whose dysfunction might limit OXPHOS and aggravate liver metabolic disorder.

As lactate acts as a permissive factor, it is plausible that abnormal hepatocytes with comprised lactate oxidative capacity may contribute to accumulated lactate in injured liver microenvironments, facilitating cellular dysfunction of their neighboring cells such as HSCs, immune cells or cancer cells. Although we have technical difficulties in deciphering the specific contribution of hepatocellular-consumed lactate for different neighboring cell types during MASH progression, our findings from in vivo efficacy studies demonstrate that AAV-TBG-mediated hepatocyte-specific ESRRA overexpression exerts essential roles in alleviating the inflammatory and fibrogenic liver lesions. Chronic exercise also recapitulates the protective effects in age-related MASLD in part attributed by the restored ESRRA signaling. Of note, aberrant H3K18la is rectified in said liver tissues. H3K18la-marked genes such as *Gdf15, Cdk1* and *Il18bp* can be impeded by ESRRA restoration, some of which are secreted factors and might provoke hepatic inflammatory and fibrotic lesions by communication with surrounding cells^41,43^. On the contrary, global knockout of ESRRA accelerates the development of diethylnitrosamine (DEN)-induced HCC because of hepatocyte necrosis and increased proinflammatory cytokines in Kupffer cells^64^. It is of great interest whether an increase in ESRRA activity in hepatocytes causes rapid depletion of local lactate in liver, which may in turn reshape the surrounding immune environment or TME.

The observed downregulation of hepatic ESRRA in MASLD/MASH suggests its suppression is a central pathogenic event, probably driven by multiple metabolic and stress signals. It has been reported that ESRRA expression is regulated by the mammalian target of rapamycin (mTOR); inhibition of mTOR promotes ubiquitin-dependent degradation of ESRRA, thereby contributing to MASLD development^65^. Furthermore, ESRRA and its co-activator PPARGC1A engage in a positive feedback loop that reinforces their mutual expression^66–68^. Disruption of this PPARGC1A–ESRRA axis can initiate a self-perpetuating cycle of metabolic dysfunction in various disease states^69,70^. Notably, a recent study demonstrated that palmitic acid-induced lipotoxicity in AML12 hepatocytes reduces ESRRA occupancy at its promoter ERRE, compromising its auto-regulatory transcription^68^. Moreover, lipid overload also inhibits PPARGC1A by endoplasmic reticulum stress-induced CHOP, leading to a mitochondrial circRNA SCAR repression, therefore facilitating steatosis-to-MASH progression^71^. Furthermore, the frequently attenuated AMPK activity and cAMP–PKA signaling in steatotic liver may drive the decline in ESRRA expression^72–75^. Although our data suggest that reduced ESRRA expression correlates with MASLD/MASH progression in multiple mouse models, its expression and associated epigenetic changes in corresponding human cohorts remain to be investigated. Nevertheless, emerging clinical and mechanistic evidence supports the relevance of ESRRA in human liver disease. In alignment with our observations, a recent study reported significantly decreased ESRRA expression in human MASH liver tissues^68^. Conversely, clinical interventions that improve metabolic liver disease appear to engage ESRRA signaling. For example, bariatric–metabolic surgery, which robustly ameliorates MASLD/MASH in patients with obesity, is associated with > 400-fold enrichment of ESRRA sites^76^. Beyond metabolic regulation, ESRRA has also been implicated in liver repair, where its activation by the tumor‑suppressor leukemia inhibitory factor receptor promotes hepatocyte growth factor expression and facilitates regeneration after partial hepatectomy or toxic injury^77^. Thus, ESRRA emerges as a multifaceted transcriptional regulator that responds to diverse hepatic stresses, and whose suppression exacerbates disease progression.

The prevalence and severity of MASLD and its progression to MASH reportedly differ between sexes, with accelerated disease progression in women after menopause^3^. Estrogen confers protection against fatty liver in ovariectomized mice and contributes to the lower incidence of HCC in women, primarily through its actions mediated by estrogen receptor alpha (ESR1)^78–81^. Hepatic ESR1 is expressed in both sexes, and its loss promotes hepatic steatosis and metabolic disturbance^81–83^. Although ESRRA does not bind to natural estrogen, it can modulate estrogen-responsive transcription by interacting with ESR1 and recognition of common binding sites^84,85^. In addition, we previously identified that ESRRA as a downstream effector of estrogen–ESR1 signaling that contributes to sex differences in MASLD susceptibility^18^. Here, we further demonstrated that estrogen deprivation in female mice alters the expression of ESRRA/PPARGC1A and LDHB as well as histone lactylation levels, which may underlie the sexually dimorphic hepatic response to metabolic stimuli such as exercise. Of note, ESRRA in the adrenal gland is recently identified to be involved in sex-specific mitochondrial changes in response to endurance training, implying its role in exercise-related sex disparity^33^. Future studies will be essential to determine whether the ESRRA-mediated metabolic–epigenetic axis exhibits sexual dimorphism during advanced MASH.

However, reduced expression or activity of LDHB represents a crucial and early event of age-related dysfunction, including the normal aging of muscle and brain^86,87^. Moreover, suppressed LDHB expression promotes the early onset of age-related hearing loss owing to mitochondrial defects and high aerobic glycolysis^88^. Importantly, reduced LDHB mRNA has been identified in human MASH samples from individuals with obesity^89^. Furthermore, LDHB suppression-mediated lactic acidosis decreases OXPHOS activity, which in turn enhances glycolysis, contributing to liver cancer progression^29^. Consistently, bioinformatic analysis of The Cancer Genome Atlas Liver Hepatocellular Carcinoma database confirms that a low LDHB-to-LDHA ratio correlates with a poor prognosis, with significantly shorter survival in comparison with the high ratio^29^. Here, we demonstrate that hepatic ablation of LDHB deteriorates the liver’s capacity to clear lactate during acute treadmill exercise and declined LDHB expression occurs in the different injured livers, which might render them susceptible to a lactate-rich microenvironment induced by various risk factors. On the contrary, our findings from ex vivo primary hepatocyte assay and in vivo studies suggest that induction of LDHB expression by endurance exercise in aged mice or ESRRA overexpression could restrain these disease-promoting alterations by elevating oxidative activity for lactate disposal. Senescent hepatocytes can induce liver steatosis owing to dysfunctional mitochondria losing the ability to metabolize efficiently^90^. However, we observed that endurance exercise enhances the expression of ESRRA/PPARGC1A and LDHB but reduces H3K18la and H3K9la, profoundly ameliorating hepatic steatosis in aged mice. By contrast, blockade of ESRRA in hepatocytes weakens hepatic response to exercise and aggravates MASH features. These findings suggest that hepatocyte ESRRA exerts beneficial effects of exercise by sustaining metabolic homeostasis to combat various deleterious stimuli. Supporting its therapeutic potential, long-term and stable restoration of hepatic ESRRA via AAV delivery in aged mice mitigates spontaneous MASLD, rectifying aberrant H3K18la accumulation and its marked gene transcripts. Collectively, our findings prove that ESRRA signaling drives gluconeogenic–epigenetic coordination in hepatocytes, which might be essential to appropriately adapt to patho- and physiological stressors such as exercise, aging, estrogen deficiency, the feed–fast cycle or malnutrition. Thus, rational targeting of the metabolism–epigenetic regulatory axis in hepatocytes represents a potential therapeutic strategy for preventing MASH in the clinic.

## Supplementary information

Supplementary Information

## Data Availability

The publicly available liver transcriptomic datasets used in this study are available in the Gene Expression Omnibus (GEO) database under accession numbers GSE66676, GSE63067, GSE164760, GSE33814, GSE89377, GSE29721, GSE126848, GSE25097, GSE77314 and GSE146049. The binding signals of ESRRA in wild-type mouse livers were obtained from the published ChIP-seq dataset GSE43638. Peaks of ESRRA binding were visualized using IGV version 2.17.4.

## References

[1] Huang, D. Q., El-Serag, H. B. & Loomba, R. Global epidemiology of NAFLD-related HCC: trends, predictions, risk factors and prevention. *Nat. Rev. Gastroenterol. Hepatol.* **18**, 223–238 (2020). 33349658 10.1038/s41575-020-00381-6 PMC8016738

[2] Kanwal, F. et al. Risk of hepatocellular cancer in patients with non-alcoholic fatty liver disease. *Gastroenterology* **155**, 1828–1837 (2018). 30144434 10.1053/j.gastro.2018.08.024 PMC6279617

[3] Klair, J. S. et al. A longer duration of estrogen deficiency increases fibrosis risk among postmenopausal women with nonalcoholic fatty liver disease. *Hepatology* **64**, 85–91 (2016). 26919573 10.1002/hep.28514 PMC4917418

[4] Younossi, Z. M., Zelber-Sagi, S., Henry, L. & Gerber, L. H. Lifestyle interventions in nonalcoholic fatty liver disease. *Nat. Rev. Gastroenterol. Hepatol.* **20**, 708–722 (2023). 37402873 10.1038/s41575-023-00800-4

[5] Ashcroft, S. P., Stocks, B., Egan, B. & Zierath, J. R. Exercise induces tissue-specific adaptations to enhance cardiometabolic health. *Cell Metab.* **36**, 278–300 (2024). 38183980 10.1016/j.cmet.2023.12.008

[6] Kraut, J. A., Ingelfinger, J. R. & Madias, N. E. Lactic acidosis. *N. Engl. J. Med.* **371**, 2309–2319 (2014). 25494270 10.1056/NEJMra1309483

[7] Schwörer, S. et al. Fibroblast pyruvate carboxylase is required for collagen production in the tumour microenvironment. *Nat. Metab.* **3**, 1484–1499 (2021). 34764457 10.1038/s42255-021-00480-x PMC8606002

[8] Daw, C. C. et al. Lactate elicits ER-mitochondrial Mg^2+^ dynamics to integrate cellular metabolism. *Cell* **183**, 474–489 (2020). 33035451 10.1016/j.cell.2020.08.049 PMC7572828

[9] Zhang, D. et al. Metabolic regulation of gene expression by histone lactylation. *Nature* **574**, 575–580 (2019). 31645732 10.1038/s41586-019-1678-1 PMC6818755

[10] Rho, H., Terry, A. R., Chronis, C. & Hay, N. Hexokinase 2-mediated gene expression via histone lactylation is required for hepatic stellate cell activation and liver fibrosis. *Cell Metab.* **35**, 1406–1423 (2023). 37463576 10.1016/j.cmet.2023.06.013 PMC11748916

[11] An, S. et al. PDHA1 hyperacetylation-mediated lactate overproduction promotes sepsis-induced acute kidney injury via Fis1 lactylation. *Cell Death Dis.* **14**, 457 (2023). 37479690 10.1038/s41419-023-05952-4 PMC10362039

[12] Chu, X. et al. Lactylated histone H3K18 as a potential biomarker for the diagnosis and predicting the severity of septic shock. *Front. Immunol.* **12**, 786666 (2022). 35069560 10.3389/fimmu.2021.786666 PMC8773995

[13] Yang, Z. et al. Lactylome analysis suggests lactylation-dependent mechanisms of metabolic adaptation in hepatocellular carcinoma. *Nat. Metab.* **5**, 61–79 (2023). 36593272 10.1038/s42255-022-00710-w

[14] Zhang, Z. et al. Serine catabolism generates liver NADPH and supports hepatic lipogenesis. *Nat. Metab.* **3**, 1608–1620 (2021). 34845393 10.1038/s42255-021-00487-4 PMC8721747

[15] Oka, S. et al. PPARα–Sirt1 complex mediates cardiac hypertrophy and failure through suppression of the ERR transcriptional pathway. *Cell Metab.* **14**, 598–611 (2011). 22055503 10.1016/j.cmet.2011.10.001 PMC3217210

[16] Ramjiawan, A., Bagchi, R. A., Albak, L. & Czubryt, M. P. Mechanism of cardiomyocyte PGC-1α gene regulation by ERRα. *Biochem. Cell Biol.* **91**, 148–154 (2013). 23668787 10.1139/bcb-2012-0080

[17] Schreiber, S. N. et al. The estrogen-related receptor α (ERRα) functions in PPARγ coactivator 1α (PGC-1α)-induced mitochondrial biogenesis. *Proc. Natl Acad. Sci. USA* **101**, 6472–6477 (2004). 15087503 10.1073/pnas.0308686101 PMC404069

[18] Yang, M. et al. Dysfunction of estrogen-related receptor alpha-dependent hepatic VLDL secretion contributes to sex disparity in NAFLD/NASH development. *Theranostics* **10**, 10874–10891 (2020). 33042259 10.7150/thno.47037 PMC7532682

[19] Huang, T. et al. Targeting adipocyte ESRRA promotes osteogenesis and vascular formation in adipocyte-rich bone marrow. *Nat. Commun.* **15**, 3769 (2024). 38704393 10.1038/s41467-024-48255-8 PMC11069533

[20] Inoue, S. -i. et al. Short-term cold exposure induces persistent epigenomic memory in brown fat. *Cell Metab.* **36**, 1764–1778 (2024). 38889724 10.1016/j.cmet.2024.05.011 PMC11305953

[21] Summermatter, S., Santos, G. & Pérez-Schindler, J. Skeletal muscle PGC-1α controls whole-body lactate homeostasis through estrogen-related receptor α-dependent activation of LDH B and repression of LDH A. *Proc. Natl Acad. Sci. USA* **110**, 8738–8743 (2013). 23650363 10.1073/pnas.1212976110 PMC3666691

[22] Liang, X. et al. Exercise inducible lactate dehydrogenase B regulates mitochondrial function in skeletal muscle. *J. Biol. Chem.* **291**, 25306–25318 (2016). 27738103 10.1074/jbc.M116.749424 PMC5207234

[23] B’chir, W. et al. Divergent role of estrogen-related receptor α in lipid- and fasting-induced hepatic steatosis in mice. *Endocrinology* **159**, 2153–2164 (2018). 29635284 10.1210/en.2018-00115

[24] Tsuchida, T. et al. A simple diet- and chemical-induced murine NASH model with rapid progression of steatohepatitis, fibrosis and liver cancer. *J Hepatol.* **69**, 385–395 (2018). 29572095 10.1016/j.jhep.2018.03.011 PMC6054570

[25] Li, F. et al. Aerobic exercise suppresses CCN2 secretion from senescent muscle stem cells and boosts muscle regeneration in aged mice. *J Cachexia Sarcopenia Muscle* **15**, 1733–1749 (2024). 38925632 10.1002/jcsm.13526 PMC11446704

[26] Chaveroux, C. et al. Molecular and genetic crosstalks between mTOR and ERRα are key determinants of rapamycin-induced nonalcoholic fatty liver. *Cell Metab.* **17**, 586–598 (2013). 23562079 10.1016/j.cmet.2013.03.003

[27] Scheiner, B. et al. Acid–base disorders in liver disease. *J Hepatol.* **67**, 1062–1073 (2017). 28684104 10.1016/j.jhep.2017.06.023

[28] Ha, T. S. et al. Lactate clearance and mortality in septic patients with hepatic dysfunction. *Am. J. Emerg. Med.* **34**, 1011–1015 (2016). 26976769 10.1016/j.ajem.2016.02.053

[29] Hong, S. M. et al. Lactic acidosis caused by repressed lactate dehydrogenase subunit B expression down-regulates mitochondrial oxidative phosphorylation via the pyruvate dehydrogenase (PDH)–PDH kinase axis. *J. Biol. Chem.* **294**, 7810–7820 (2019). 30923124 10.1074/jbc.RA118.006095 PMC6514637

[30] Xia, H., Dufour, C. R. & Giguère, V. ERRα as a bridge between transcription and function: role in liver metabolism and disease. *Front. Endocrinol.* **10**, 206 (2019). 10.3389/fendo.2019.00206 PMC6459935 31024446

[31] Sung, K.-C. et al. Effect of exercise on the development of new fatty liver and the resolution of existing fatty liver. *J. Hepatol.* **65**, 791–797 (2016). 27255583 10.1016/j.jhep.2016.05.026

[32] Thyfault, J. P. & Rector, R. S. Exercise combats hepatic steatosis: potential mechanisms and clinical implications. *Diabetes* **69**, 517–524 (2020). 32198195 10.2337/dbi18-0043 PMC7085252

[33] Amar, D. et al. Temporal dynamics of the multi-omic response to endurance exercise training. *Nature* **629**, 174–183 (2024). 38693412 10.1038/s41586-023-06877-w PMC11062907

[34] Yoon, J. C. et al. Control of hepatic gluconeogenesis through the transcriptional coactivator PGC-1. *Nature* **413**, 131–138 (2001). 11557972 10.1038/35093050

[35] Miller, R. A. & Birnbaum, M. J. Glucagon: acute actions on hepatic metabolism. *Diabetologia* **59**, 1376–1381 (2016). 27115415 10.1007/s00125-016-3955-y

[36] Taniguchi, C. M. et al. Cross-talk between hypoxia and insulin signaling through Phd3 regulates hepatic glucose and lipid metabolism and ameliorates diabetes. *Nat. Med.* **19**, 1325–1330 (2013). 24037093 10.1038/nm.3294 PMC4089950

[37] Kaymak, I. et al. Carbon source availability drives nutrient utilization in CD8^+^ T cells. *Cell Metab.* **34**, 1298–1311 (2022). 35981545 10.1016/j.cmet.2022.07.012 PMC10068808

[38] Hui, S. et al. Glucose feeds the TCA cycle via circulating lactate. *Nature* **551**, 115–118 (2017). 29045397 10.1038/nature24057 PMC5898814

[39] Hui, S. et al. Quantitative fluxomics of circulating metabolites. *Cell Metab.* **32**, 676–688 (2020). 32791100 10.1016/j.cmet.2020.07.013 PMC7544659

[40] Giguère, V. Transcriptional control of energy homeostasis by the estrogen-related receptors. *Endocr. Rev.* **29**, 677–696 (2008). 18664618 10.1210/er.2008-0017

[41] Zhou, T. et al. IL-18BP is a secreted immune checkpoint and barrier to IL-18 immunotherapy. *Nature* **583**, 609–614 (2020). 32581358 10.1038/s41586-020-2422-6 PMC7381364

[42] Yang, L. et al. Nucleolin lactylation contributes to intrahepatic cholangiocarcinoma pathogenesis via RNA splicing regulation of MADD. *J. Hepatol.* **81**, 651–666 (2024). 38679071 10.1016/j.jhep.2024.04.010

[43] L’Homme, L. et al. Adipose tissue macrophage infiltration and hepatocyte stress increase GDF-15 throughout development of obesity to MASH. *Nat. Commun.* **15**, 7173 (2024). 39169003 10.1038/s41467-024-51078-2 PMC11339436

[44] Wu, C. X. et al. Blocking CDK1/PDK1/β-catenin signaling by CDK1 inhibitor RO3306 increased the efficacy of sorafenib treatment by targeting cancer stem cells in a preclinical model of hepatocellular carcinoma. *Theranostics* **8**, 3737–3750 (2018). 30083256 10.7150/thno.25487 PMC6071527

[45] Ni, H.-M. et al. Nrf2 promotes the development of fibrosis and tumorigenesis in mice with defective hepatic autophagy. *J. Hepatol.* **61**, 617–625 (2014). 24815875 10.1016/j.jhep.2014.04.043 PMC4143992

[46] Chen, X. et al. Lactylation-driven FTO targets CDK2 to aggravate microvascular anomalies in diabetic retinopathy. *EMBO Mol. Med.* **16**, 294–318 (2024). 38297099 10.1038/s44321-024-00025-1 PMC10897304

[47] Xiong, J. et al. Lactylation-driven METTL3-mediated RNA m^6^A modification promotes immunosuppression of tumor-infiltrating myeloid cells. *Mol. Cell* **82**, 1660–1677 (2022). 35320754 10.1016/j.molcel.2022.02.033

[48] Zhang, C. et al. H3K18 lactylation potentiates immune escape of non-small cell lung cancer. *Cancer Res.* **84**, 3589–3601 (2024). 39137401 10.1158/0008-5472.CAN-23-3513

[49] Liu, Q. et al. Glycogen accumulation and phase separation drives liver tumor initiation. *Cell* **184**, 5559–5576 (2021). 34678143 10.1016/j.cell.2021.10.001

[50] Li, F. et al. FBP1 loss disrupts liver metabolism and promotes tumorigenesis through a hepatic stellate cell senescence secretome. *Nat. Cell Biol.* **22**, 728–739 (2020). 32367049 10.1038/s41556-020-0511-2 PMC7286794

[51] Gou, D. et al. Gluconeogenic enzyme PCK1 supports *S*-adenosylmethionine biosynthesis and promotes H3K9me3 modification to suppress hepatocellular carcinoma progression. *J. Clin. Invest.* **133**, e161713 (2023). 37166978 10.1172/JCI161713 PMC10313362

[52] Gallage, S. et al. A 5:2 intermittent fasting regimen ameliorates NASH and fibrosis and blunts HCC development via hepatic PPARα and PCK1. *Cell Metab.* **36**, 1371–1393 (2024). 38718791 10.1016/j.cmet.2024.04.015

[53] Petersen, K. F., Dufour, S., Mehal, W. Z. & Shulman, G. I. Glucagon promotes increased hepatic mitochondrial oxidation and pyruvate carboxylase flux in humans with fatty liver disease. *Cell Metab.* **36**, 2359–2366 (2024). 39197461 10.1016/j.cmet.2024.07.023 PMC11612994

[54] Perry, R. J. et al. Glucagon stimulates gluconeogenesis by INSP3R1-mediated hepatic lipolysis. *Nature* **579**, 279–283 (2020). 32132708 10.1038/s41586-020-2074-6 PMC7101062

[55] Chen, M. et al. Cdo1-Camkk2-AMPK axis confers the protective effects of exercise against NAFLD in mice. *Nat. Commun.* **14**, 8391 (2023). 38110408 10.1038/s41467-023-44242-7 PMC10728194

[56] Saks, V., Buler, M., Aatsinki, S.-M., Izzi, V. & Hakkola, J. Metformin reduces hepatic expression of SIRT3, the mitochondrial deacetylase controlling energy metabolism. *PLoS ONE* **7**, e49863 (2012). 23166782 10.1371/journal.pone.0049863 PMC3500349

[57] Liu, D., Benlhabib, H. & Mendelson, C. R. cAMP enhances estrogen-related receptor α (ERRα) transcriptional activity at the SP-A promoter by increasing its interaction with protein kinase A and steroid receptor coactivator 2 (SRC-2). *Mol. Endocrinol.* **23**, 772–783 (2009). 19264843 10.1210/me.2008-0282 PMC2691680

[58] Bonnelye, E., Reboul, P., Duval, N., Cardelli, M. & Aubin, J. E. Estrogen receptor–related receptor α regulation by interleukin-1β in prostaglandin E2- and cAMP-dependent pathways in osteoarthritic chondrocytes. *Arthritis Rheum.* **63**, 2374–2384 (2011). 21506092 10.1002/art.30398

[59] Knudsen, N. H. et al. Interleukin-13 drives metabolic conditioning of muscle to endurance exercise. *Science* **368**, eaat3987 (2020). 32355002 10.1126/science.aat3987 PMC7549736

[60] Perry, M.-C., Dufour, C. R., Tam, I. S., B’Chir, W. & Giguère, V. Estrogen-related receptor-α coordinates transcriptional programs essential for exercise tolerance and muscle fitness. *Mol. Endocrinol.* **28**, 2060–2071 (2014). 25361393 10.1210/me.2014-1281 PMC5414781

[61] Barrès, R. et al. Acute exercise remodels promoter methylation in human skeletal muscle. *Cell Metab.* **15**, 405–411 (2012). 22405075 10.1016/j.cmet.2012.01.001

[62] Chen, J., Wu, Y., Hao, W., You, J. & Wu, L. Non-canonical hepatic androgen receptor mediates glucagon sensitivity in female mice through the PGC1α/ERRα/mitochondria axis. *Cell Rep.* **28**, 115118 (2025). 10.1016/j.celrep.2024.115188 39792556

[63] Hong, W. et al. PGC-1α loss promotes mitochondrial protein lactylation in acetaminophen-induced liver injury via the LDHB–lactate axis. *Pharmacol. Res.* **205**, 107228 (2024). 38810904 10.1016/j.phrs.2024.107228

[64] Hong, E.-J., Levasseur, M.-P., Dufour, C. R., Perry, M.-C. & Giguère, V. Loss of estrogen-related receptor α promotes hepatocarcinogenesis development via metabolic and inflammatory disturbances. *Proc. Natl Acad. Sci. USA* **110**, 17975–17980 (2013). 24127579 10.1073/pnas.1315319110 PMC3816417

[65] Chaveroux, C. et al. Molecular and genetic crosstalks between mTOR and ERRalpha are key determinants of rapamycin-induced nonalcoholic fatty liver. *Cell Metab.* **17**, 586–598 (2013). 23562079 10.1016/j.cmet.2013.03.003

[66] Liu, D., Zhang, Z. & Teng, C. T. Estrogen-related receptor-gamma and peroxisome proliferator-activated receptor-gamma coactivator-1 alpha regulate estrogen-related receptor-alpha gene expression via a conserved multi-hormone response element. *J. Mol. Cell. Cardiol.* **34**, 473–487 (2005). 10.1677/jme.1.01586 15821111

[67] Laganiere, J. et al. A polymorphic autoregulatory hormone response element in the human estrogen-related receptor alpha (ERRalpha) promoter dictates peroxisome proliferator-activated receptor gamma coactivator-1 alpha control of ERR alpha expression. *J. Biol. Chem.* **279**, 18504–18510 (2004). 14978033 10.1074/jbc.M313543200

[68] Tripathi, M. et al. Esrra regulates Rplp1-mediated translation of lysosome proteins suppressed in metabolic dysfunction-associated steatohepatitis and reversed by alternate day fasting. *Mol. Metab.* **87**, 101997 (2024). 39032642 10.1016/j.molmet.2024.101997 PMC11327444

[69] Sihag, S., Cresci, S., Li, A. Y., Sucharov, C. C. & Lehman, J. J. PGC-1 alpha and ERR alpha target gene downregulation is a signature of the failing human heart. *J. Mol. Cell. Cardiol.* **46**, 201–212 (2009). 19061896 10.1016/j.yjmcc.2008.10.025 PMC2681265

[70] Xu, J. et al. The pro-inflammatory cytokine IL6 suppresses mitochondrial function via the gp130-JAK1/STAT1/3-HIF1alpha/ERR alpha axis. *Cell Rep.* **44**, 115403 (2025). 40056415 10.1016/j.celrep.2025.115403

[71] Zhao, Q. et al. Targeting mitochondria-located circRNA SCAR alleviates NASH via reducing mROS output. *Cell* **183**, 76–93 (2020). 32931733 10.1016/j.cell.2020.08.009

[72] Sopariwala, D. H. et al. Estrogen-related receptor alpha is an AMPK-regulated factor that promotes ischemic muscle revascularization and recovery in diet-induced obese mice. *FASEB Bioadv.* **4**, 602–618 (2022). 36089981 10.1096/fba.2022-00015 PMC9447423

[73] Hu, X. et al. AMP activated protein kinase-alpha 2 regulates expression of estrogen-related receptor-alpha, a metabolic transcription factor related to heart failure development. *Hypertension* **58**, 696–703 (2011). 21825219 10.1161/HYPERTENSIONAHA.111.174128 PMC3182261

[74] Zhao, P. et al. AR. An AMPK–caspase-6 axis controls liver damage in nonalcoholic steatohepatitis. *Science* **367**, 652–660 (2020). 32029622 10.1126/science.aay0542 PMC8012106

[75] Liu, D., Benlhabib, H. & Mendelson, C. R. cAMP enhances estrogen-related receptor alpha (ERR alpha) transcriptional activity at the SP-A promoter by increasing its interaction with protein kinase A and steroid receptor coactivator 2 (SRC-2). *Mol. Endocrinol.* **23**, 772–783 (2009). 19264843 10.1210/me.2008-0282 PMC2691680

[76] Ahrens, M. et al. DNA methylation analysis in nonalcoholic fatty liver disease suggests distinct disease-specific and remodeling signatures after bariatric surgery. *Cell Metab.* **18**, 296–302 (2013). 23931760 10.1016/j.cmet.2013.07.004

[77] Deng, Y. et al. LIFR regulates cholesterol-driven bidirectional hepatocyte–neutrophil cross-talk to promote liver regeneration. *Nat. Metab.* **6**, 1756–1774 (2024). 39147934 10.1038/s42255-024-01110-y PMC11498095

[78] Zhu, L. et al. Estrogen treatment after ovariectomy protects against fatty liver and may improve pathway-selective insulin resistance. *Diabetes* **62**, 424–434 (2013). 22966069 10.2337/db11-1718 PMC3554377

[79] Naugler, W. E. et al. Gender disparity in liver cancer due to sex differences in MyD88-dependent IL-6 production. *Science* **317**, 121–124 (2007). 17615358 10.1126/science.1140485

[80] Tian, Y., Hong, X., Xie, Y., Guo, Z. & Yu, Q. 17 Beta-estradiol (E_2_) upregulates the ER alpha/SIRT1/PGC-1 alpha signaling pathway and protects mitochondrial function to prevent bilateral oophorectomy (OVX)-induced nonalcoholic fatty liver disease (NAFLD). *Antioxidants* **12**, 2100 (2023). 38136219 10.3390/antiox12122100 PMC10740447

[81] Allard, C. et al. Activation of hepatic estrogen receptor-alpha increases energy expenditure by stimulating the production of fibroblast growth factor 21 in female mice. *Mol. Metab.* **22**, 62–70 (2019). 30797705 10.1016/j.molmet.2019.02.002 PMC6437689

[82] Park, C. J. et al. Genetic rescue of nonclassical ER alpha signaling normalizes energy balance in obese ER alpha-null mutant mice. *J. Clin. Invest.* **121**, 604–612 (2011). 21245576 10.1172/JCI41702 PMC3026715

[83] Khristi, V. et al. Disruption of ESR1 alters the expression of genes regulating hepatic lipid and carbohydrate metabolism in male rats. *Mol. Cell. Endocrinol.* **490**, 47–56 (2019). 30974146 10.1016/j.mce.2019.04.005

[84] Deblois, G. & Giguere, V. Oestrogen-related receptors in breast cancer: control of cellular metabolism and beyond. *Nat. Rev. Cancer* **13**, 27–36 (2013). 23192231 10.1038/nrc3396

[85] Zheng, Z. Z. et al. Nuclear receptor ESRRA promotes ER alpha-positive breast cancer through dual action on super enhancers and promoters to regulate gene transcriptional programs. *Sci. Bull.* **70**, 3822–3839 (2025). 10.1016/j.scib.2025.09.044 41111053

[86] Washington, T. A., Healey, J. M., Thompson, R. W., Lowe, L. L. & Carson, J. A. Lactate dehydrogenase regulation in aged skeletal muscle: regulation by anabolic steroids and functional overload. *Exp. Gerontol.* **57**, 66–74 (2014). 24835193 10.1016/j.exger.2014.05.003

[87] Ross, J. M. et al. High brain lactate is a hallmark of aging and caused by a shift in the lactate dehydrogenase A/B ratio. *Proc. Natl Acad. Sci. USA* **107**, 20087–20092 (2010). 21041631 10.1073/pnas.1008189107 PMC2993405

[88] Tian, C. et al. Suppressed expression of LDHB promotes age-related hearing loss via aerobic glycolysis. *Cell Death Dis* **11**, 375 (2020). 32415082 10.1038/s41419-020-2577-y PMC7229204

[89] de Mello, V. D. et al. Human liver epigenetic alterations in non-alcoholic steatohepatitis are related to insulin action. *Epigenetics* **12**, 287–295 (2017). 28277977 10.1080/15592294.2017.1294305 PMC5398766

[90] Ogrodnik, M. et al. Cellular senescence drives age-dependent hepatic steatosis. *Nat. Commun.* **8**, 15691 (2017). 28608850 10.1038/ncomms15691 PMC5474745

