# Supplementary material for: Hepatocyte estrogen-related receptor α modulates a gluconeogenic–epigenetic crosstalk counteracting MASLD/MASH progression
Source: Exp Mol Med. 2026 May 8;58(5):1536–55. doi: 10.1038/s12276-026-01707-1 (PMC13233843; doi:10.1038/s12276-026-01707-1)
Supplement: Supplementary file 1 — Supplementary Information [file 12276_2026_1707_MOESM1_ESM.pdf]

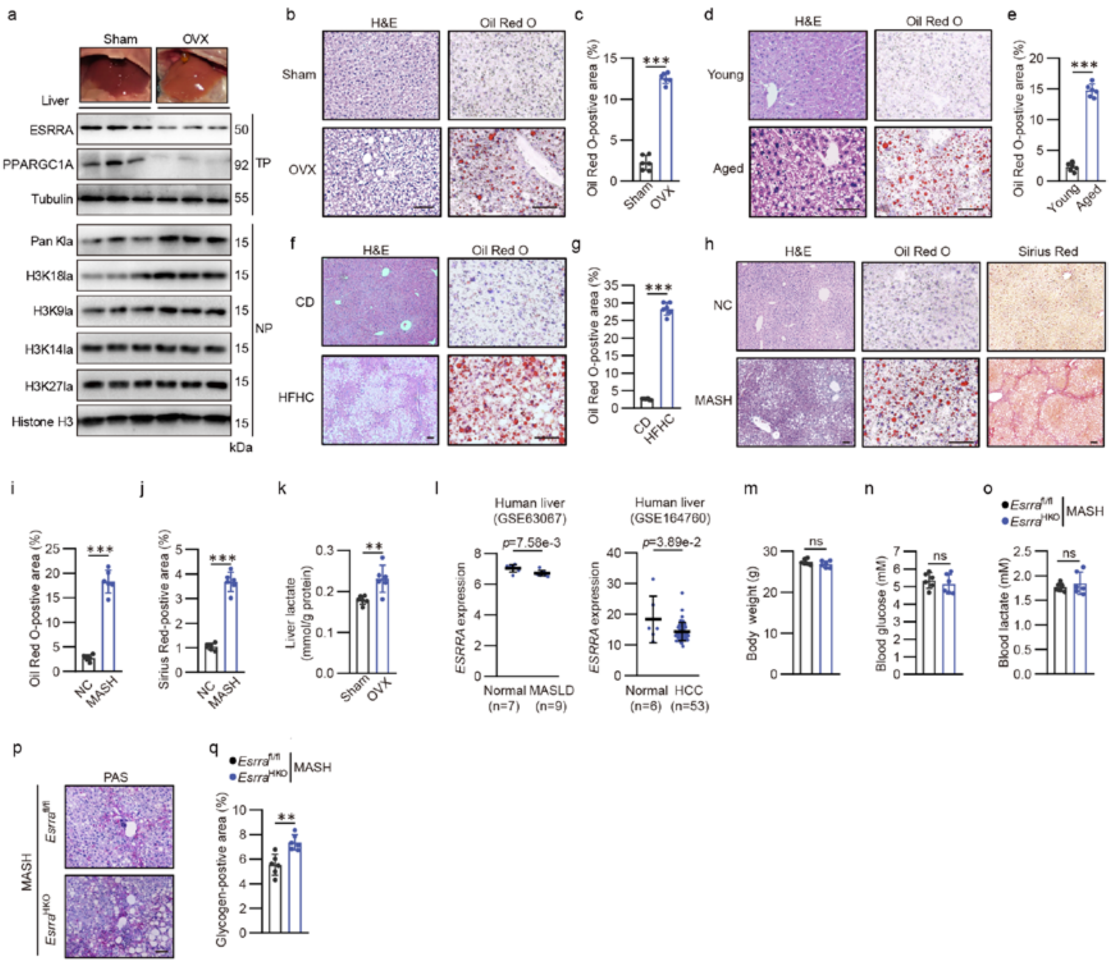

Supplementary Fig. 1 Declined hepatic ESRRA/PPARGC1A expression is associated with MAFLD/MASH in diverse murine models and patients.

a Gross morphology of livers and indicated protein levels from female mice underwent either sham or OVX operation for 8 week. **b-g** Representative H&E and Oil Red O staining of liver sections from female mice received either sham or OVX operation (**b**), young (3-month-old) versus aged (25-month-old) male mice (**d**), and female mice fed a chow diet (CD) or a high-fat/high-cholesterol (HFHC) diet for 28 weeks (**f**). Quantification of Oil Red O stained areas (**c, e, g**) from livers as in (**b**, **d**, **f**), respectively. Scale bar, 50 µm. n = 6 mice. **h** Representative images of H&E, Oil Red O and Sirius Red staining of liver sections from control MASH mice that received a GAN diet and CCl_4 for 12 weeks. Scale bar, 50 µm. **i, j** Quantification of Oil Red O (**i**) and Sirius Red (**j**) stained areas in (**h**). n = 6 mice. **k** Hepatic lactate levels in (**a**). n = 6 mice. **l** Relative *ESRRA* mRNA levels of human normal, MASLD and HCC livers from GSE63067 and GSE164760 datasets. **m-o** Body weight (**m**), blood glucose (**n**)

and lactate (**o**) in *Esrra*$^{fl/fl}$ and *Esrra*^{HKO} MASH mice. n = 6 mice. **p** Representative images of PAS staining of liver sections from *Esrra*$^{fl/fl}$ and *Esrra*^{HKO} MASH mice. Scale bar, 50 μm. **q** Quantification of glycogen-positive areas in (**p**). n = 6 mice. Results were expressed as mean ± SD. Statistical analysis was calculated using two-tailed Student's t-test (**c, e, g, i-k, m-o, q**) and Wald test by DESeq2 (**l**). *$p < 0.05$, **$p < 0.01$ and ***$p < 0.001$.

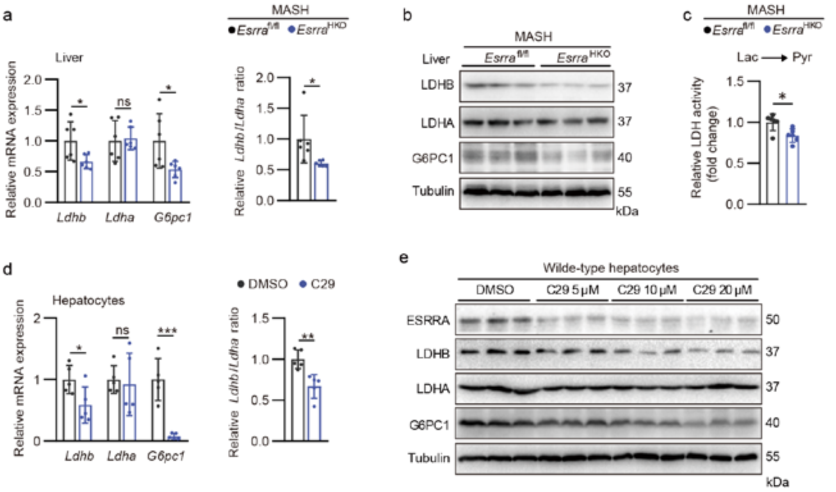

Supplementary Fig. 2 mRNA and protein expression of LDHB and G6PC1 in ESRRA-ablation MASH liver and C29-treated hepatocytes.

a The mRNA levels of *Ldhb*, *Ldha*, *G6pc1*, and *Ldhb*/*Ldha* ratio in livers from *Esrra*$^{fl/fl}$ and *Esrra*^{HKO} MASH mice. n = 6 mice. **b** Hepatic protein levels of LDHB, LDHA and G6PC1. **c** Relative lactate-to-pyruvate LDH activity in *Esrra*$^{fl/fl}$ and *Esrra*^{HKO} MASH mice. n = 6 mice. **d** mRNA expression of *Ldhb*, *Ldha*, *G6pc1*, and *Ldhb*/*Ldha* ratio in wild-type hepatocytes treated with 20 μM C29 for 24h. n = 5. **e** Protein levels of ESRRA, LDHB, LDHA and G6PC1 in wild-type hepatocytes treated with different doses of C29 for 24h. Results were expressed as mean ± SD. Statistical analysis was calculated using two-tailed Student's t-test (**a,c,d**). *$p < 0.05$, **$p < 0.01$ and ***$p < 0.001$.

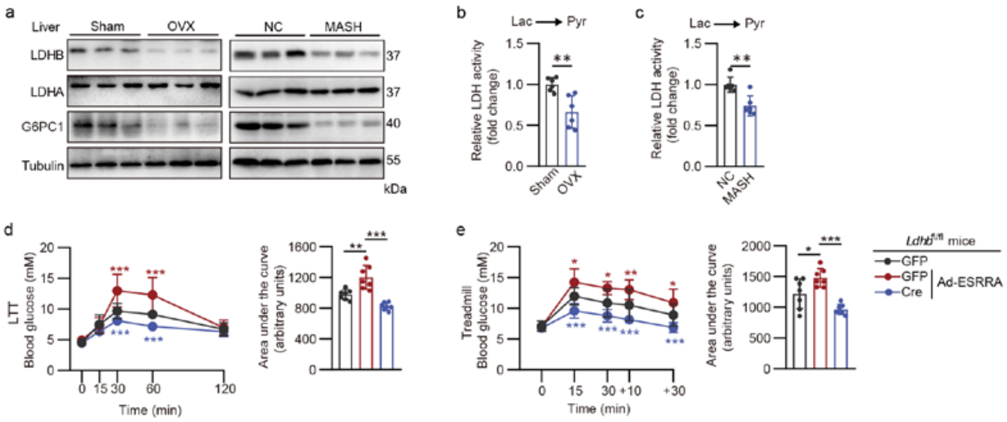

Supplementary Fig. 3 Reduced hepatic protein of gluconeogenic enzymes LDHB and G6PC1 are associated with OVX or MASH and hepatic ESRRA facilitates gluconeogenesis dependent on LDHB in mice.

a Western blot analysis of LDHB, LDHA and G6PC1 proteins in liver lysates from the indicated mice. **b, c** Relative lactate-to-pyruvate LDH activity in OVX (**b**), MASH (**c**) and control mice livers, respectively. n = 6 mice. **d**,**e** Blood glucose excursion curves and the corresponding AUC during LTT (**d**) and treadmill exercise (**e**) were analyzed in *Ldhb*$^{fl/fl}$ mice infected with Ad-GFP, and Ad-ESRRA with or without Ad-Cre. n = 7 mice. Statistical analysis was calculated using two-tailed Student's t-test (**b, c**), Statistical analysis was calculated using two-way ANOVA with Tukey's post hoc test (**d, e**). *p < 0.05, **p < 0.01 and ***p < 0.001.

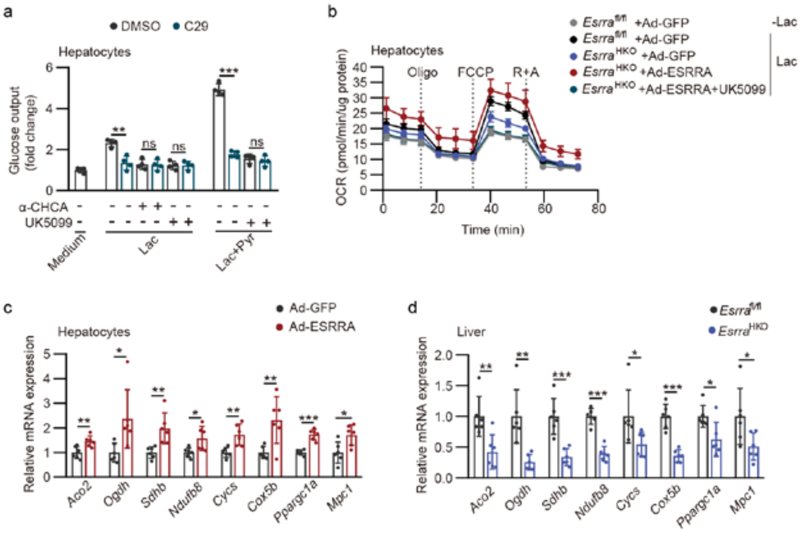

Supplementary Fig. 4 ESRRA facilities lactate oxidation and OXPHOS-associated gene expression in hepatocytes.

a Glucose production supported by Lac with or without Pry in wild-type hepatocytes was examined with treatment of vehicle, 5 mM α-CHCA or 5 μM UK5099 for 6h. n = 4. **b** OCR assay in primary hepatocytes was measured after acute exposure to lactate. The hepatocytes isolated from *Esrra*$^{fl/fl}$ and *Esrra*^{HKO} mice were treated with UK5099 or vehicle for 6h after infection with Ad-ESRRA or Ad-GFP. n = 4. **c**,**d** qPCR analysis of mRNA levels of OXPHOS-associated genes in hepatocytes infected with Ad-ESRRA or Ad-GFP (**c**), or in *Esrra*$^{fl/fl}$ and *Esrra*^{HKO} mice livers (**d**). n = 6. Statistical analysis was calculated using two-tailed Student's t-test (**a**, **c, d**). *$p < 0.05$, **$p < 0.01$ and ***$p < 0.001$.

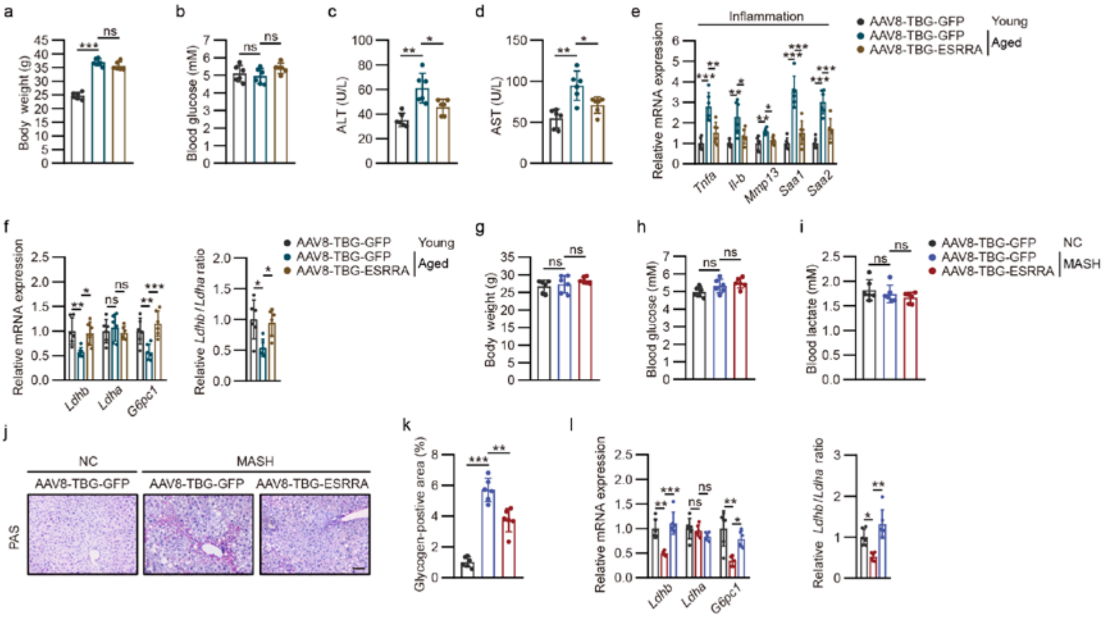

Supplementary Fig. 5 Metabolic features in age-related MASLD or MASH mice treated with AAV-TBG-ESRRA.

a-d Body weight (**a**), blood glucose (**b**), ALT (**c**) and AST (**d**) levels in young (3-month-old) and aged (18-month-old) male mice infected with AAV8-TBG-ESRRA or AAV8-TBG-GFP. n = 6 mice. **e** Hepatic mRNA levels of genes invovled in inflammation. n = 6 mice**. f** Hepatic mRNA levels of *Ldhb*, *Ldha* and *G6pc1*, and *Ldhb*/*Ldha* ratio. n = 6 mice. **g-i** Body weight (**g**), blood glucose (**h**) and lactate (**i**) levels in normal and MASH mice infected with AAV8-TBG-ESRRA or AAV8-TBG-GFP. n = 6 mice. **j** Representative images of PAS staining in liver sections. Scale bar, 50 μm. **k** Quantification of glycogen-positive areas in (**j**). n = 6 mice. **l** mRNA expression of *Ldhb*, *Ldha* and *G6pc1*, and *Ldhb*/*Ldha* ratio in mice livers. n = 6 mice. Statistical analysis was calculated using two-way ANOVA with Tukey's post hoc test (**a-i, k, l**). **p* < 0.05, ***p* < 0.01 and ****p* < 0.001.

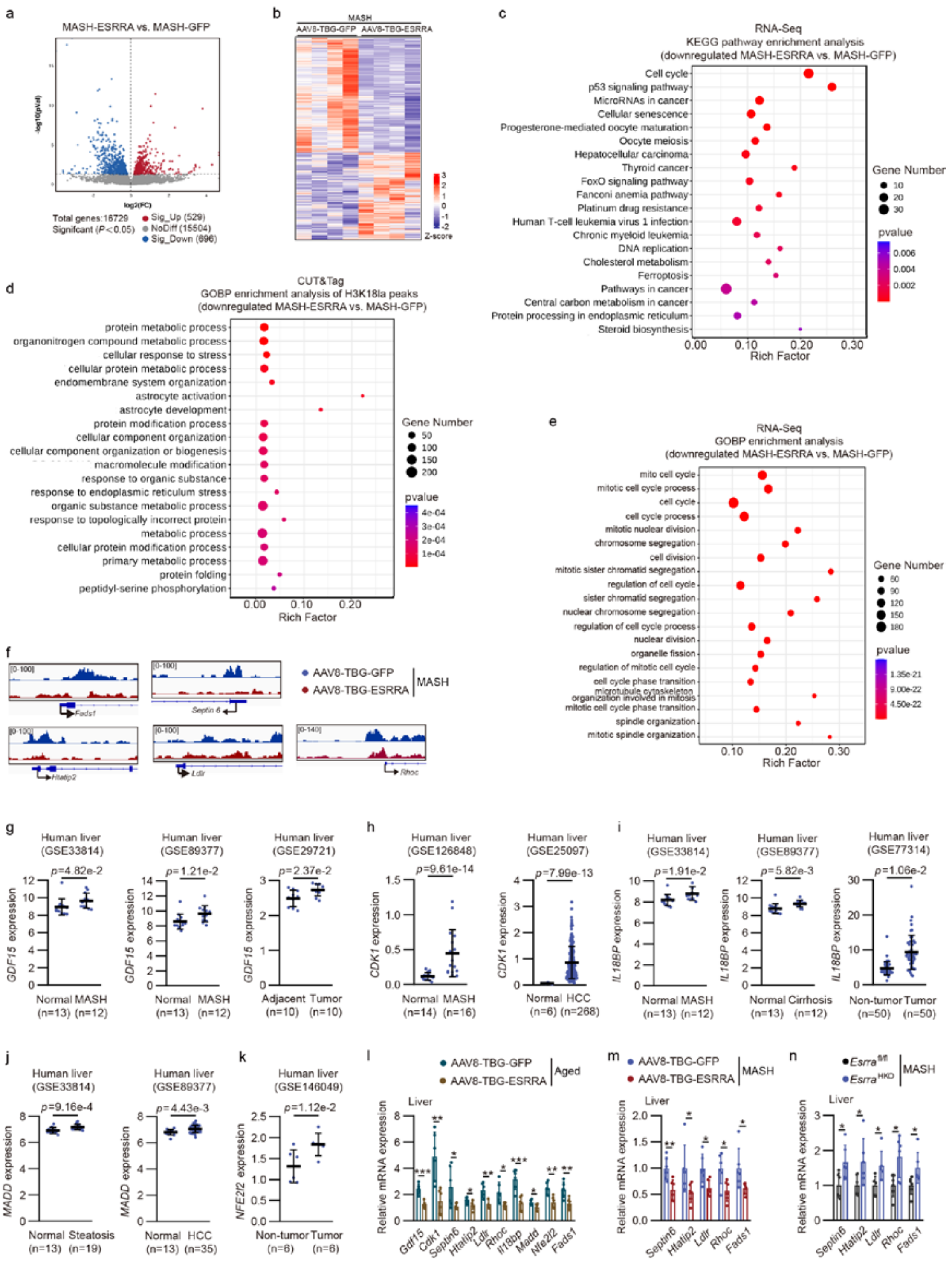

Supplementary Fig. 6 Genomic analysis of transcriptional consequences of ESRRA and H3K18la involved in MASH progression.

a, b Volcano plot (**a**) and heatmap (**b**) depicting the differential expressed genes in liver between MASH-GFP group and MASH-ESRRA group. Differential expressed genes were determined using DESeq2 analysis ($p < 0.05$). **c** KEGG enrichment analysis of downregulated expressed genes in MASH-ESRRA group from RNA-seq

data. **d** Gene Ontology Biological Process (GOBP) enrichment analysis of H3K18la target genes with reduced H3K18la modification in MASH-ESRRA group, based on CUT-Tag data. **e** GOBP enrichment analysis of downregulated expressed genes in MASH-ESRRA group from RNA-Seq data. **f** Genome browser tracks of CUT-Tag signal at the H3K18la target genes including *Fads1*, *Septin6*, *Htatip2*, *Ldlr* and *Rhoc* from CUT&Tag data. **g-k** Database analysis of relative mRNA expression of *GDF15* (**g**), *CDK1* (**h**), *IL18BP* (**i**), *MADD* (**j**), *NFE2L2* (**k**) in livers of healthy individuals and indicated patients. **l** Hepatic mRNA levels of indicated genes from aged (18-month-old) mice administered AAV8-TBG-ESRRA or AAV8-TBG-GFP. n = 6 mice. **m** Hepatic mRNA levels of indicated genes in livers in MASH-GFP group and MASH-ESRRA group were examined using RT-qPCR. n = 6 mice. **n** Hepatic mRNA expression of indicated genes in livers from $Esrra^{fl/fl}$ and $Esrra^{HKO}$ MASH mice by qPCR analysis. n = 6 mice. Results were expressed as mean ± SD. Statistical analysis was calculated using Wald test by DESeq2 (**g-k**), two-tailed Student's t-test (**l-n**). *$p < 0.05$, **$p < 0.01$ and ***$p < 0.001$.

Supplementary Table 1. Primers used for genotyping

Primer name	Sequence 5'---3'
Esrra-P1	CTGGAGAAAACTGAGGAGAGGA
Esrra-P2	ATGCTCCTAGTTCCATTCCAAA
Cre-P1	TGCAAACATCACATGCACAC
Cre-P2	TTGGCCCCTTACCATAACTG
Cre-P3	GAAGCAGAAGCTTAGGAAGATGG

Supplementary Table 2. Primers for promoter vectors

Primer name	Sequence 5'---3'
Ldhb-WT-luc forward	CCGGGTACCGGCTGGATGAGACAAAGAGCTAA
Ldhb-WT-luc reverse	CCGAAGCTTCCACAACACACACCCTGATGTT
G6pc1-WT-luc forward	CGGGGTACCCACTCTCCGGACTGGTTCAT
G6pc1-WT-luc reverse	CCGCTCGAGGGTTGGCCTTGATCCCTCTG
G6pc1-mutA-luc forward	TCTGTGATCGCTGATTTCAGAGACACCTTC
G6pc1-mutA-luc reverse	GAAGGTGTCTCTGAAATCAGCGATCACAGA
G6pc1-mutB-luc forward	TTAATAACTTAAAATTTCACTTCCGGCAGT
G6pc1-mutB-luc reverse	ACTGCCGGAAGTGAAATTTTAAGTTATTAA
G6pc1-mutC-luc forward	CAGGGCTGGATTGATTTACAGACTGAATCC
G6pc1-mutC-luc reverse	GGATTCAGTCTGTAAATCAATCCAGCCCTG

Supplementary Table 3. Primers for ChIP-qPCR

Primer name	Sequence 5'---3'
G6cp1 siteA forward	CACTCTCCGGACTGGTTCAT
G6cp1 siteA reverse	AGGGGATGGTTCCAGATAGGA
G6cp1 siteB forward	GACAACAAAGCCCTACTGCTG
G6cp1 siteB reverse	ACTCTTGCAAGGGCATCTGTT
G6cp1 siteC forward	TTGAGTCCAAAGATCAGGGCT
G6cp1 siteC reverse	GGCCTTGATCCCTCTGCTATC
Ldhb forward	CACAAGATGGAGACCGTACAGA
Ldhb reverse	TCGGCTCGTTCAATCTCCTTC
Ppargc1a forward	TTGACTGTGTGGAAAGTAGAGCCC
Ppargc1a reverse	CTCCCACAAAGAAACACTAATTTTT

Supplementary Table 4. Primers for RT-qPCR

Primer name	Sequence 5’---3’
Ppargc1a forward	TATGGAGTGACATAGAGTGTGCT
Ppargc1a reverse	CCACTTCAATCCACCCAGAAAG
Ldhb forward	CATTGCGTCCGTTGCAGATG
Ldhb reverse	GGAGGAACAAGCTCCCGTG
Ldha forward	TGTCTCCAGCAAAGACTACTGT
Ldha reverse	GACTGTACTTGACAATGTTGGGA
G6pc1 forward	TTACCAAGACTCCCAGGACTG
G6pc1 reverse	GAGCTGTTGCTGTAGTAGTCG
Fasn forward	GGCATCATTGGGCACTCCTT
Fasn reverse	GCTGCAAGCACAGCCTCTCT
Hmgcr forward	AGTCAGTGGGAACTATTGCAC
Hmgcr reverse	TTACGTCAACCATAGCTTCCG
Scd1 forward	TTCTTGCGATACACTCTGGTGC
Scd1 reverse	CGGGATTGAATGTTCTTGTCGT
Srebp1 forward	GCAGCCACCATCTAGCCTG
Srebp1 reverse	CAGCAGTGAGTCTGCCTTGAT
Acad11 forward	CGCCTTGGACCTGGAAGAAT
Acad11 reverse	TTCAAGGTCAGCAAGCGGAT
Acadm forward	AGGGTTTAGTTTTGAGTTGACGG
Acadm reverse	CCCCGCTTTTGTCATATTCCG
Acoxl forward	CTGCCAAGGGACTCCAGAGCAGCT
Acoxl reverse	GACATGGACACATCCACCATGCAG
Acat2 forward	ACTCTCTTCCAGCCATCTTTCA
Acat2 reverse	ATAGGTGGTTTCGTGGATGC
Timp1 forward	GCAACTCGGACCTGGTCATAA
Timp1 reverse	CGGCCCGTGATGAGAAACT
Col1a1 forward	ATCGGTCATGCTCTCTCCAAACCA
Col1a1 reverse	ACTGCAACATGGAGACAGGTCAGA
Serpine1 forward	ATCAATGACTGGGTGGAAAGGC
Serpine1 reverse	GTTGAACTTGTTGCTCTGAGCC
Mmp13 forward	ATCCAGCTAAGACACAGCAAGCCA
Mmp13 reverse	TGGAGCACAAAGGAGTGGTCTCAA
Saa1 forward	GGACATGAGGACACCATTGC
Saa1 reverse	GTAGGAAGAAGCCCAGACCC
Saa2 forward	TGGCTGGAAAGATGGAGACAA
Saa2 reverse	AAAGCTCTCTCTTGCATCACTG
Aco2 forward	ATCGAGCGGGGAAAGACATAC
Aco2 reverse	TGATGGTACAGCCACCTTAGG
Ogdh forward	AGGGCATATCAGATACGAGGG
Ogdh reverse	CTGTGGATGAGATAATGTCAGCG

Continue on next page

Primer name	Sequence 5’---3’
Sdhb forward	AGCTACTGGTGGAACGGAGA
Sdhb reverse	GCAGCGGTAGACAGAGAAGG
Ndufb8 forward	TTGCCTGCAAACTCTGTGAG
Ndufb8 reverse	CTCCACAATGGCATCAACAG
Cycs forward	CCAAATCTCCACGGTCTGTTC
Cycs reverse	ATCAGGGTATCCTCTCCCCAG
Cox5b forward	GATGAGGAGCAGGCTACTGG
Cox5b reverse	TGCAGCCCACTATTCTCTTG
Mpc1 forward	TTATCAGTGGGCGGATGACAT
Mpc1 reverse	GCTGTACCTTGTAGGCAAATCTC
Il-b forward	AATGCCACCTTTTGACAGTGATG
Il-b reverse	AGCTTCTCCACAGCCACAAT
Tnfa forward	AGGGTCTGGGCCATAGAACT
Tnfa reverse	CCACCACGCTCTTCTGTCTAC
Col3a1 forward	AAACAGCAAATTCACTTACAC
Col3a1 reverse	ACCCCCAATGTCATAGG
Elovl7 forward	ACCATCATCCTGGGCCTCTA
Elovl7 reverse	CAGCCAGACATCACAAACTCATA
18S forward	TAAGTCCCTGCCCTTTGTACACA
18S reverse	GATCCGAGGGCCTCACTAAAC
β-actin forward	GGCTGTATTCCCCTCCATCG
β-actin reverse	CCAGTTGGTAACAATGCCATGT
Gdf15 forward	CCTGGTCTGGGGATACTGAG
Gdf15 reverse	AGCAGGAACAGCAGGAACC
Nfe2l2 forward	GCCCACATTCCCAAACAAG
Nfe2l2 reverse	CCAGAGAGCTATTGAGGGACTG
Fads1 forward	GAAGAAGCACATGCCATACAACC
Fads1 reverse	TCCGCTGAACCACAAAATAGAAA
Cdk1 forward	GTCCGTCGTAACCTGTTGAG
Cdk1 reverse	TGACTATATTTGGATGTCGAAG
Septin6 forward	GAGGAGCTGAAGATCCGAAGAGTA
Septin6 reverse	CCTCGTTTTCAACCTGCACAGTC
Htatip2 forward	TTTTCCAAAGTAACGCTCAT
Htatip2 reverse	CAGGCAACAGAATCCAACAT
Ldlr forward	TGACTCAGACGAACAAGGCTG
Ldlr reverse	ATCTAGGCAATCTCGGTCTCC
Rhoc forward	GAGGCAAGATGAGCATACCAGGAGA
Rhoc reverse	GAGAATGGGACAGCCCCTCCGGCG
Il18bp forward	ACATCTGCACCTCAGACAACT
Il18bp reverse	TGGGAGGTGCTCAATGAAGGAACCA
Madd forward	GGCCCTGACATTTGCTCTTCC
Madd reverse	CATCACAGACATGGAGAGTGCA
